# Unique signalling connectivity of FGFR3-TACC3 oncoprotein revealed by quantitative phosphoproteomics and differential network analysis

**DOI:** 10.18632/oncotarget.22048

**Published:** 2017-10-25

**Authors:** Benedetta Lombardi, Paul Ashford, Aurelio A. Moya-Garcia, Aleksander Rust, Mark Crawford, Sarah V. Williams, Margaret A. Knowles, Matilda Katan, Christine Orengo, Jasminka Godovac-Zimmermann

**Affiliations:** ^1^ Proteomics and Molecular Cell Dynamics, Center for Nephrology, School of Life and Medical Sciences, University College London, London NW3 2PF, United Kingdom; ^2^ Institute of Structural and Molecular Biology, Division of Biosciences, University College London, London WC1E 6BT, United Kingdom; ^3^ Section of Molecular Oncology, Leeds Institute of Molecular Medicine, St James’s University Hospital, Leeds LS9 7TF, United Kingdom

**Keywords:** FGFR-TACC-fusion, cancer, quantitative phosphoproteomics, signaling pathways, network analysis

## Abstract

The FGFR3-TACC3 fusion is an oncogenic driver in diverse malignancies, including bladder cancer, characterized by upregulated tyrosine kinase activity. To gain insights into distinct properties of FGFR3-TACC3 down-stream signalling, we utilised telomerase-immortalised normal human urothelial cell lines expressing either the fusion or wild-type FGFR3 (isoform IIIb) for subsequent quantitative proteomics and network analysis. Cellular lysates were chemically labelled with isobaric tandem mass tag reagents and, after phosphopeptide enrichment, liquid chromatography-high mass accuracy tandem mass spectrometry (LC-MS/MS) was used for peptide identification and quantification. Comparison of data from the two cell lines under non-stimulated and FGF1 stimulated conditions and of data representing physiological stimulation of FGFR3 identified about 200 regulated phosphosites. The identified phosphoproteins and quantified phosphosites were further analysed in the context of functional biological networks by inferring kinase-substrate interactions, mapping these to a comprehensive human signalling interaction network, filtering based on tissue-expression profiles and applying disease module detection and pathway enrichment methods. Analysis of our phosphoproteomics data using these bioinformatics methods combined into a new protocol—Disease Relevant Analysis of Genes On Networks (DRAGON)—allowed us to tease apart pathways differentially involved in FGFR3-TACC3 signalling in comparison to wild-type FGFR3 and to investigate their local phospho-signalling context. We highlight 9 pathways significantly regulated only in the cell line expressing FGFR3-TACC3 fusion and 5 pathways regulated only by stimulation of the wild-type FGFR3. Pathways differentially linked to FGFR3-TACC3 fusion include those related to chaperone activation and stress response and to regulation of TP53 expression and degradation that could contribute to development and maintenance of the cancer phenotype.

## INTRODUCTION

The Fibroblast Growth Factors (FGFs) and their receptors (FGFRs) regulate multiple biological processes and their dysfunction has been implicated in a range of developmental disorders and a variety of cancer types [1–3]. The growing number of FGFR inhibitors currently in cancer clinical trials follow numerous pre-clinical studies that established these receptors as attractive therapeutic targets [4, 5]. Over-expression and activating point-mutations are common causes of FGFR signalling dysregulation, but FGFR fusions have also been discovered in diverse tumours [6, 7]. The FGFR3-TACC3 fusion was initially discovered in glioblastoma [8] and bladder cancer [9]. Subsequently, recurrent FGFR-TACC gene fusions have been identified in a range of malignancies that include non-small cell lung cancer, oral and head neck squamous carcinoma, cervical carcinoma and triple negative breast cancer, with a frequency typically between 1% and 4% [10–13]. Nevertheless, taken across different cancer types, FGFR-TACC fusions, with FGFR3-TACC3 being by far most frequent, have now emerged as one of the most recurrent chromosomal translocations. Furthermore, these fusions have been linked to oncogene addiction and confer particular sensitivity to targeted agents; data on clinical response, although limited, are also promising [14, 15].

The size of FGFR3-TACC3 fusion proteins vary. They combine an FGFR3 portion lacking only a small part at the C-terminus and variable portions of Transforming Acid Coiled-Coil containing protein 3 (TACC3); for example, a fusion found in a bladder cancer cell line RT112, that is also frequently observed in other tumours, comprises amino acids 1–760 of FGFR3 (IIIb isoform) fused in-frame to amino acids 648–838 of TACC3 [9, 12]. Centrosomal TACC3 is involved in regulation of mitosis [16] and the protein contains a coiled-coiled domain that is incorporated in all fusion proteins, presumably contributing to constitutive dimerisation [17].

The effects of FGFR3-TACC3 expression on cellular functions are still largely unknown. Initial studies based on exogenous expression suggest that the functional effects seem to be dependent on the cell background. For example, as previously reported for several activating FGFR3 point-mutations, NIH3T3 cells expressing FGFR3-TACC3 show a transformed phenotype that was not observed in telomerase-immortalised normal human urothelial cells (TERT-NHUC) used in studies of initiation and development of bladder cancer, where major changes attributed to mutated FGFR3 include increased survival and proliferation to high cell density [9, 18, 19]. Nevertheless, the fusion is invariably constitutively phosphorylated when expressed in a range of different cell types which, in turn, has been linked to various (but underexplored) changes in cell signalling [8, 9, 14, 17, 20–24]. There is also evidence that FGF ligands further enhance fusion-mediated signalling [20]. Another distinct property suggested for FGFR3-TACC3 is a change in subcellular localisation manifested in a higher portion of the fusion (compared to WT FGFR3) being present in the nucleus and, in dividing cells, in mitotic spindle poles [8, 24]; this, together with differences in activation mechanisms, could result in phosphorylation of novel targets rather than just an enhancement of physiological, agonist-driven FGFR signalling.

To date, a comprehensive study exploring the effects of FGFR3-TACC3 on cellular signalling has not been performed. The present work aims to present a first overview of the effects of FGFR3-TACC3 expression in urothelial cells and highlight potential differences from physiological FGFR3 signalling. We use quantitative mass spectrometry (MS)-based phosphoproteomics methods that have emerged in recent years as a powerful approach to study phosphorylation dynamics in cell cultures and tissues [25]. As previously described, we adapted these methods to allow analysis of small amounts of TERT-NHUC cells [26]. To further develop existing approaches for data analysis [27–29], we assemble pathway and network bioinformatics tools in a novel protocol that reveals new signalling links unique to the FGFR3-TACC3 fusion.

## RESULTS

### Initial characterisation of selected cellular responses

**Figure 1:**
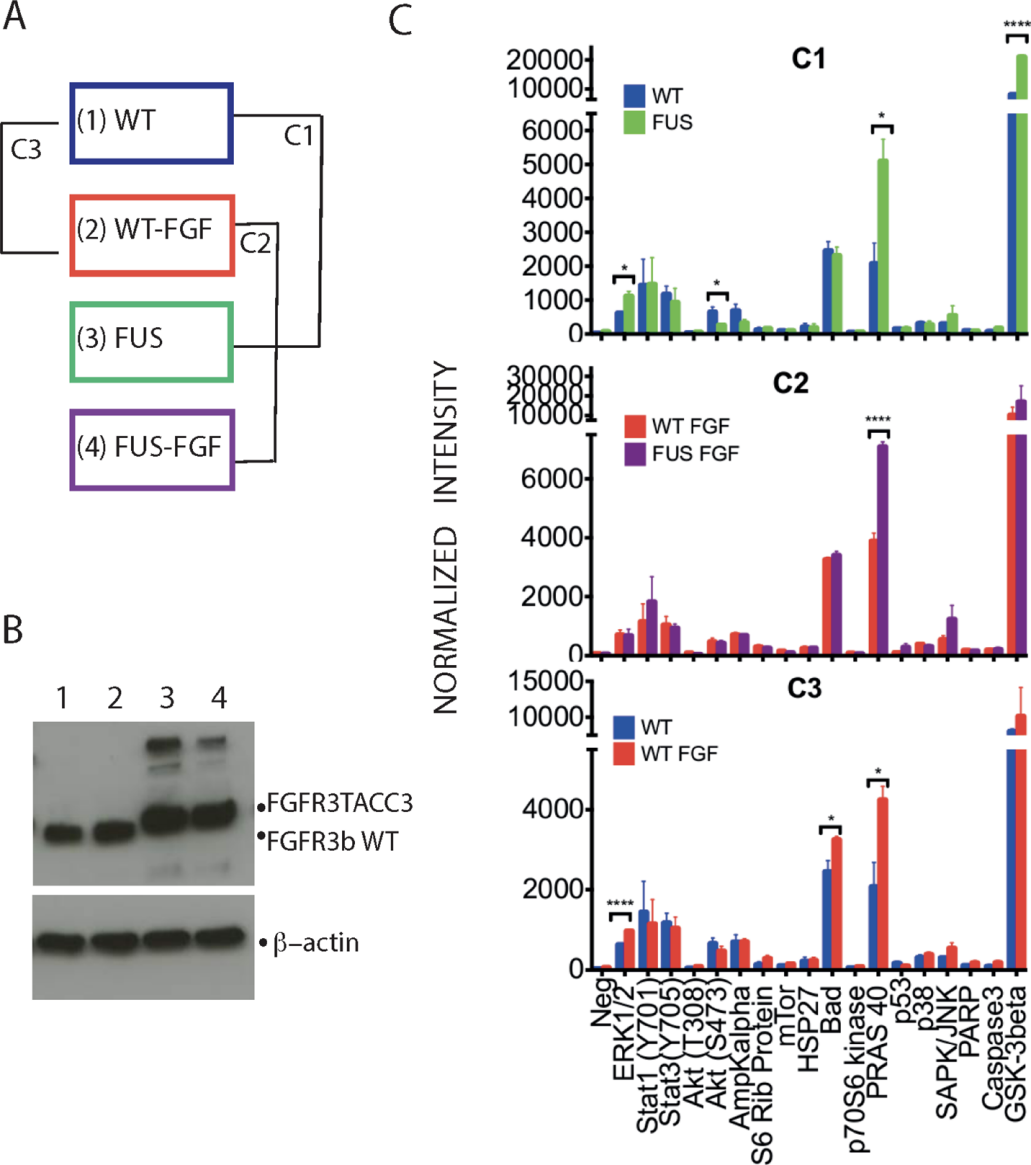
Phosphorylation profiles of selected intracellular signalling proteins allows initial comparison of WT and FUS and their responses to FGF stimulation (**A**) Two TERT-NHUC cells lines, stably expressing either the WT FGFR3 (IIIb) or FGFR3-TACC3 fusion, were analysed without or following stimulation by 100 ng/ml FGFR1 for 10 min (in both cases 100 IU/ml heparin was included in incubation medium for 10 min). The four resulting experimental conditions have been designated as WT (1), WT-FGF (2), FUS (3) and FUS-FGF (4). Further comparisons of data generated from these conditions were between WT and FUS (comparison 1; C1), WT-FGF and FUS-FGF (comparison 2; C2) and WT and WT-FGF (comparison 3; C3). (**B**) Western blotting with anti-FGFR3 antibody to detect WT FGFR3 or FGFR3-TACC3 fusion in cell lysates from four experimental conditions described in (A): WT (1), WT-FGF (2), FUS (3) and FUS-FGF (4) (top panel). β-actin was used as a loading control (bottom panel). (**C**) Antibody array analysis comprising indicated proteins and their phospho-sites performed using cell lysates from four conditions, defined and compared as outlined in (A). Comparisons cover WT and FUS (C1) (top panel), WT-FGF and FUS-FGF (C2) (middle panel) and WT and WT-FGF (C3) (bottom panel). Data are shown as average signals from two independent experiments on two different biological replicates (number of stars: significantly changing phosphosites, unpaired Student’s *t*-test, *p* ≤ 0.05. Error bars: S.E.M.). See Supplementary Figure 1 for further information about the antibody array and analyses.

Our previous comparison between FGFR3-TACC3 fusion protein (corresponding to boundaries found in the urothelial cancer cell line RT112 and a number of clinical samples) and WT FGFR3 (IIIb) in TERT-NHUC cell lines included only limited assessments of downstream signaling; for example, in the absence of FGF1 stimulation, phosphorylation of ERK1/2 was detected only in cells expressing FGFR3-TACC3 fusion [9]. For the extensive MS-based phosphoproteome analysis described here, we used the same cell lines under non-stimulated and FGF1 stimulated conditions. As outlined in Figure 1A, we refer to these experimental conditions as WT, WT-FGF, FUS and FUS-FGF and comparisons WT vs FUS as C1, WT-FGF vs FUS-FGF as C2 and WT vs WT-FGF as C3.

**Figure 2:**
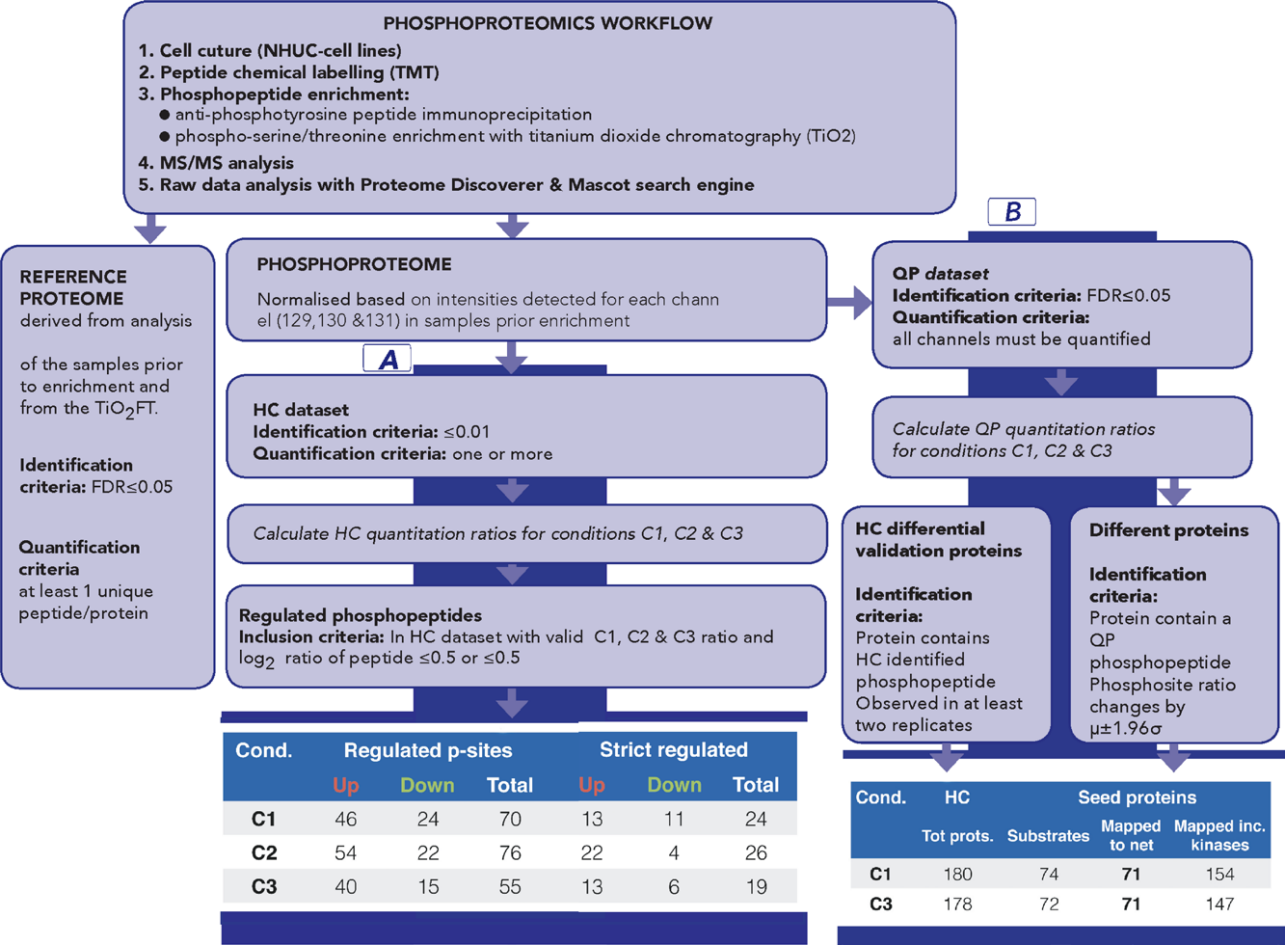
Overview of phosphoproteomics workflow for data generation and analysis The workflow provides both phospho and reference proteome datasets. **Part A** of the data analysis workflow, based on High Confidence (HC) quantitative proteomics dataset, has been designed to identify the most significantly regulated phosphosites between C1, C2 and C3. Number of regulated and sites for each comparison is summarised (part A, last panel); strict regulated sites additionally require >1 replicate and that μ±1.96σ does not include 1. **Part B** of the data analysis workflow has been designed to generate seeds for subsequent network analysis; it is based on Quantitative Proteomics (QP) dataset [QP requires all channels to be quantified but with a less stringent FDR (5%)] to allow a broad pool of initial seeds while HC dataset is used to filter pathway results. Number of proteins for C1 and C3 is shown (part B, last panel). See also Supplementary Tables 1– 6 and Supplementary Figure 2 (for replicate correlations) and Supplementary Figure 4 (for comparison of HC and QP datasets).

The different conditions were initially characterized for the expression of WT FGFR3 and FGFR3-TACC3 fusion (Figure 1B). Subsequently, an antibody-based array for a set of specific phosphoproteins that play pivotal roles in intracellular signaling (Figure 1C and Supplementary Figure 1) was used to detect significant phosphoprotein changes for comparisons (C1, C2 and C3). Comparison C1 (WT vs FUS) revealed higher phosphoprotein content for FUS, notably for pERK (T202/Y204), pPRAS 40 (T246) and GSK-3beta (S9), with pPRAS 40 (T246) also showing differential increase in comparison to C2 (WT-FGF vs FUS-FGF) (Figure 1C, top and middle panels). Following FGF stimulation in cells expressing WT, pERK1/2 (T202/Y204), pPRAS (T246) and pBad (S112) were all up-regulated (C3; Figure 1C, bottom panel). Data from the antibody-based array therefore confirm an increase in phosphorylation in FUS (C1) and FGF stimulation of WT cells (C3) as well as the consistency of the biological replicates (Figure 1C). It is also interesting that the significant increase in pGSK3β (S9) and decrease in pAKT (S473) in C1 was not observed in C3 while the increase in pBad was only seen in C3. Although covering only a few example proteins, this suggests that some phospho-signalling changes in FUS versus WT may involve pathway changes rather than simply modulation of physiological FGF stimulation, at least under conditions used here. Such a possibility was further explored *via* MS-based phosphoproteomic and subsequent bioinformatics analyses; a flowchart integrating these strategies is shown in Figure 2 with further detail described in Supplementary Information (Supplementary Tables 1–6 and Supplementary Figures 2 and 4).

### Quantitative phosphoproteomic analysis

**Figure 3:**
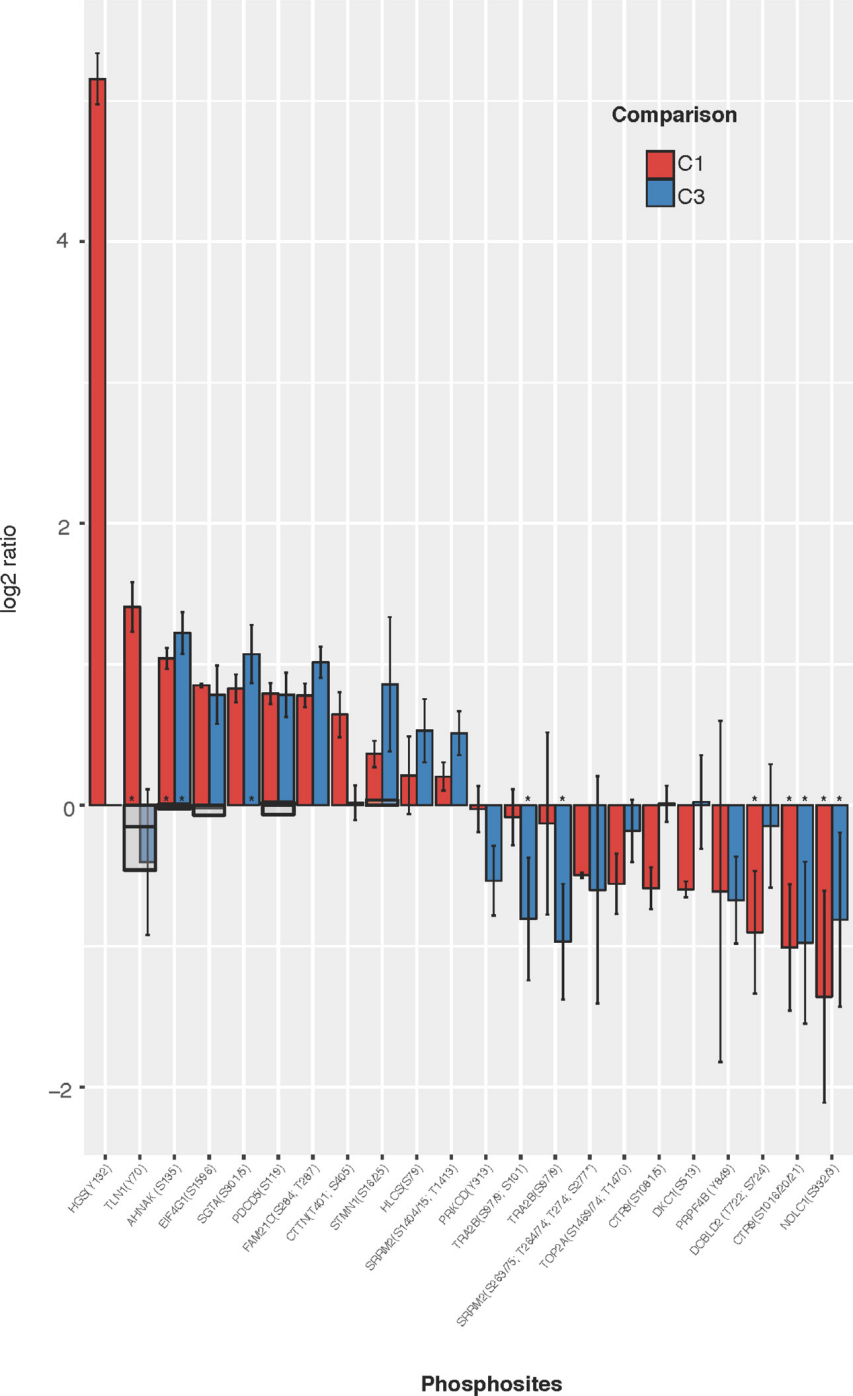
Most significantly regulated phosphosites identified from comparisons of WT vs FUS (C1) and WT vs WT-FGF (C3) HC phosphopeptides are shown where mean C1 or C3 ratio shows both a substantial (|log_2_(comparison ratio)| > 0.5) and a significant (using +/–1.96σ) change. Phosphosites observed in only one replicate are excluded. Error bars show 2 standard deviations (log corrected). Where available, reference proteome levels are indicated as grey boxes. Peptides also passing SigB significance shown as (^*^).

The most prominently regulated phosphosites were identified by applying the workflow for sample labelling, phosphopeptide enrichment and phosphoproteomics analysis to obtain a High Confidence (HC) quantitative phosphoproteomics dataset for the four conditions (WT, WT-FGF, FUS and FUS-FGF) and calculated mean comparison ratios C1 (WT vs FUS), C2 (WT-FGF vs FUS-FGF) and C3 (WT vs WT-FGF) using the observed replicates for each phosphosite (Figure 2, part A, Supplementary Table 2 and Supplementary Figure 2). Using the HC dataset the significantly regulated phosphosites for each condition have been defined as up-regulated: log_2_(p-sites in C1, 2 or 3) ≥ 0.5 and down-regulated: log_2_(p-sites in C1, 2 or 3) ≤ –0.5. This selection comprises 70 phosphopeptides (46 up-regulated and 24 down-regulated) in C1, 76 phosphopeptides (54 up-regulated and 22 down-regulated) in C2 and 55 phosphopeptides (40 up-regulated and 15 down-regulated) in C3 (Figure 2, part A and Supplementary Table 4). We further filtered this set by removing peptides that were only observed in a single replicate, or where the variance (using 1.96σ) was too high to explicitly designate a phosphosite as up or down regulated. Regulated phosphosite significance was additionally tested using SigB (*p* ≤ 0.05) [30] (Figure 2, part A and Supplementary Table 5). The resulting set of the most significant HC regulated phosphosites for comparisons C1 and C3 is shown in Figure 3. Where available, corresponding proteome changes show that it is the phosphosites that are modulated, not protein abundance (Figure 3). In addition, Western blotting and quantitative phosphoproteomics gave similar changes for C1 and C3 (Supplementary Figure 3).

The most clearly differentially regulated phosphosites (those changed in only one comparison) in C1 are up-regulation of HGS(Y132), TLN1(Y70) and CTTN(T401/S405), with down-regulation of CTR9(S1081/5) and DKC1(S513). For C3, there is down-regulation of phosphosites PRKCD (Y313) and TRA2B(S97/9/101).

To better understand the cellular signalling context of these variable phosphosites we analysed them in the context of biological pathways and functional interaction networks. We obtained sets of network “seed” proteins for a comparison by identifying those with at least one variable phosphosite (comparison ratio, using *1.96σ*, greater or less than one). As not all comparisons can be calculated using the HC dataset (for example, if a particular phosphopeptide is identified in all conditions except WT, then we can calculate C2, but not C1 or C3), we used a Quantitative Phosphoproteomics (QP) dataset comprising only phosphopeptides where reporter ions for all four experimental conditions were observed with <5% FDR (Figure 2, part B and Supplementary Table 6). Between HC and QP 74% of proteins are commonly identified (Supplementary Figure 4), with QP providing a broader set of seeds for exploration of network differences beyond just those most stringently observed in HC. However, the HC dataset is subsequently employed as a quality control filter to retain only the most relevant and confidently identified biological pathways (Figure 2, part B). The network seed proteins derived from the QP dataset and their overlap with HC proteins are shown in Supplementary Table 6_._

### Differential pathway analysis

**Figure 4:**
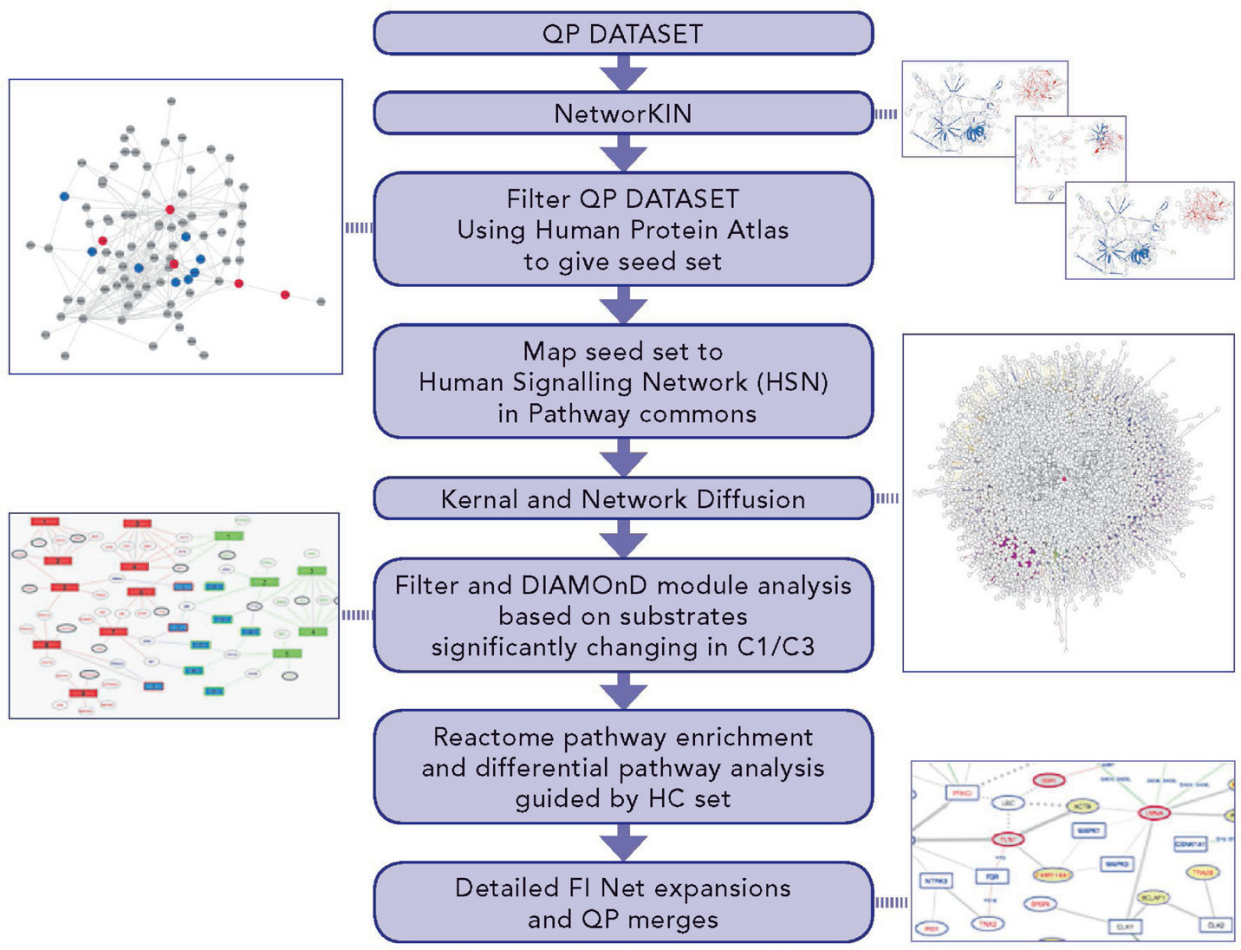
Differential pathway and network analysis process - DRAGON protocol - to identify networks specific to either FUS or WT signaling DRAGON uniquely expands and filters protein interaction networks. Depicted multiple steps cover: (i) expansion of observed altered phosphosites to include predicted kinases; (ii) filtering by tissue expression; (iii) seeding and expansion of a general PPI network (the HSN) through a graph-kernel to create an effective network focused on each comparison; (iv) expansion of the proteins in each comparison using a module detection algorithm to give sets of proteins significantly implicated in signalling events; (v) enrichment in module sets for Reactome pathways; (vi) filtering by subtraction of pathway lists to remove identical hits in each comparison; (vii) filtering differential pathways to exclude those without at least one HC protein and (viii) detailed functional network expansion around specific pathways.

Our analysis workflow (DRAGON) has been designed to identify differences in signalling pathways under different cellular conditions and applied here to examine: (a) to what extent downstream signalling in FUS is constitutively activated and similar to physiological WT FGF signalling and, importantly, (b) to also identify specific differences in signalling pathways between them (see Methods and Supplementary Experimental Procedures). We therefore focused on the C1 and C3 comparisons of the MS-based datasets. The workflow summarised in Figure 4 expands the scope of the phosphosite regulation analysis so far considered (Figures 1 and 3) to include predicted kinase-substrate interactions, broader functional interactions on a human signalling network and significant differences in biological pathways implicated in each of the conditions. Figure 4 summarises the bioinformatics data sources, tools and methods applied to identify differences in pathways between C1 and C3 (for full detail see Supplementary Experimental Procedures).

Differential pathway analysis was performed at the protein-level, i.e. only the distinct proteins were mapped onto the network. Where multiple phosphosites were identified for a protein it was considered a “substrate-seed” if any phosphosite met the criteria for significant up or down regulation; up/down regulation is defined using the QP dataset for those phosphopeptides with quantitation ratios (for a given comparison) strictly greater than or less than 1 using the standard deviation limits of *1.96σ* (thus phosphopeptides observed only once are excluded). For C1, the 74 substrate-seeds identified led to 86 possible kinase-seeds, of which AKT1 and GSK3B were also substrates. For C3, the 72 substrate seeds gave 83 possible kinases, of which EGFR, GSK3β, PTK6 and PRKCD were also substrates (Supplementary Table 6).

Seed proteins for C1 and C3 were separately mapped onto a human signalling network, from which proteins had been removed (filtered) that were unlikely to be expressed in either normal or tumour urothelial cells (for detailed network construction see Supplementary Information). From the 158 C1 seeds, 154 were mapped, excluding FYN (filtered), HDGFRP2, SRPR and kinase HCK (not in network). For the 151 C3 seeds, 147 were mapped, excluding FYN (filtered), HCK, MST4 and MAP2K6 (not in network). We extended the mapped seeds to include related proteins on the filtered network using a disease-module-finding algorithm DIAMOnD [31], returning an additional 200 proteins for each condition. DIAMOnD attempts to find proteins significantly related to seeds using a searching/scoring algorithm that finds more distantly connected proteins than methods using module-detection by clustering using local network topology; the resulting protein sets have been shown to correlate better with those known to be disease-associated [32].

The final DIAMOnD-enriched lists of proteins for C1 (*n* = 354) and C3 (*n* = 347) were independently tested for Reactome pathway enrichment, resulting in 322 pathways for C1 and 309 for C3 (FDR 1%). Differential pathways—those that highlight key processes differing between FUS and WT-FGF signalling—were found by considering the total sets of ReactomeIDs returned and applying the following three set operations (RID=Reactome pathway ID):

$$\mathrm{unique}\;C1=\{{C1}_{\mathrm{RID}}\}-\{{C3}_{\mathrm{RID}}\};$$

$$\mathrm{unique}\;C3=\{{C3}_{\mathrm{RID}}\}-\{{C1}_{\mathrm{RID}}\};$$

$$\mathrm{shared}\;C1\&C3=\{{C1}_{\mathrm{RID}}\}\cap \{{C3}_{\mathrm{RID}}\}$$

We found 23 pathways unique to C1, 10 unique to C3 and 299 shared—i.e. found in both C1 and C3. To ensure high-quality pathways, we removed any that did not contain at least one protein from our HC dataset in the list of protein ‘hits’ used to identify the pathway, resulting in nine pathways in C1 and five in C3. To find pathways common to C1 and C3 with evidence of differential regulation (i.e., due to different proteins identified as part of the same pathway between the two conditions), we identified pathways having 25% more protein hits in one condition as being more significantly associated with that condition. Of the 299 common pathways, high-quality C1-C3-shared pathways included three with preferential C1 involvement and six with preferential C3 involvement.

**Figure 5:**
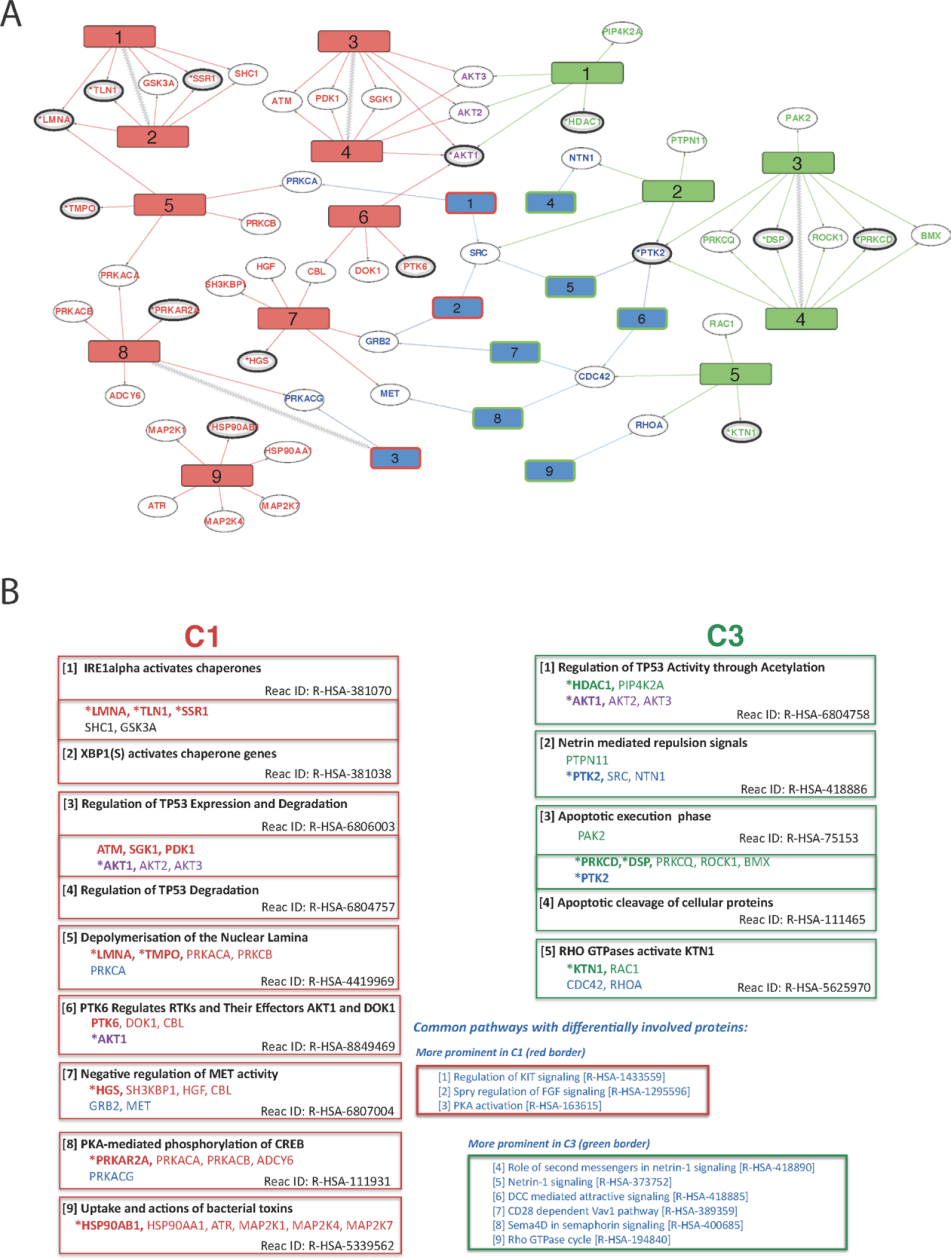
Overview of differentially regulated pathways derived from comparisons of WT *vs* FUS (C1) and WT *vs* WT-FGF (C3) (**A**) Diagram showing overall connectivity. Reactome pathways uniquely affected in C1 (left, red numbered rectangles) or C3 (right, green numbered rectangles) are shown along with links to protein ‘hits’ on each pathway (ellipses). Pathways common to both conditions, but with differential involvement of proteins are shown in blue, with red or green borders indicating preferential C1 or C3 involvement respectively. Pathways and proteins that link the two conditions are shown if there is evidence of differential involvement and hidden otherwise. Pathways directly connected in the Reactome hierarchy (i.e. related) are connected with zigzags. Proteins are coloured according to their respective pathways (red: C1-only, green: C3-only, purple: linker between C1 & C3 and blue: part of a common pathway that may also link C1 or C3). Confidence of protein identification indicated by reference to HC (^*^, bold ellipse) or QP (bold ellipse) datasets. **(B)** Summary of proteins and the Reactome pathway descriptions for each differentially regulated Reactome pathway. Red: C1-only pathway / protein; green: C3-only; purple: protein linking C1-only and C3-only pathways; blue: pathway common to both C1 and C3 but with differentially involved proteins. Protein confidence levels: (^*^bold) - High, (bold) - Medium and (non-bold) - from network analyses. The pathways are numbered by increasing FDR, except where two have been merged.

The results of the pathway analysis are summarised using a network-style diagram in Figure 5, which shows pathways specific to each condition (C1-only, C3-only, along with associated proteins), the differentially regulated common pathways (C1-C3-shared) and the proteins that connect them. This analysis helps distil the complexity of the phosphoproteome datasets into sets of specific biological processes likely to underlie differential signalling and focus on the most important aspects underlying the oncogenic potential of the FGFR3-TACC3 fusion found in many cancer types. From a total of 2050 human Reactome pathways, Figure 5 displays the 23 most significant for differential regulation between C1 and C3 and, where found, proteins common to multiple pathways.

### Differential regulation in C1 and C3

For C3-only, the strongest evidence for active pathways not found in C1 is the pair formed from (green) pathways [3] - ‘Apoptotic execution phase’ and [4] - ‘Apoptotic cleavage of cellular proteins’ (Figure 5). Involvement of these apoptosis pathways only in the WT-FGF condition and not in FUS is strongly supported by three HC proteins in common: PTK2, DSP and PRKCD. Additionally, PTK2 is shared with pathway [2] - ‘Netrin mediated repulsion signals’ and the C1-C3-shared differentially regulated pathways [5] - ‘Netrin-1 signaling’ and 6 - ‘DCC mediated attractive signaling’ (DCCs form netrin-1 receptors) (Figure 5). Loss of Netrin-1 signalling is implicated in the inhibition of p53-dependent apoptosis [33]. Additionally, C3-only pathway [1] - ‘Regulation of TP53 Activity through Acetylation’ provides further evidence that the regulation of TP53 activity and execution of apoptosis occur only in the WT-FGF condition and are not present in FUS. A detailed functional interaction network centred on ‘Apoptotic execution phase’ and ‘Apoptotic cleavage of cellular proteins’, including phospho-signalling, is shown in Supplementary Figure 5.

The strongest evidence for C1-only processes outlined in Figure 5 are the chaperone activation pathways (1 & 2), each having three HC proteins identified: LMNA, TLN1 and SSR1. Additionally, via LMNA, these chaperone pathways appear to be connected to involvement in depolymerisation of the nuclear lamina (C1-only, pathway 5), which contains the HC protein TMPO. A further pathway in C1-only (9 - ‘uptake and actions of bacterial toxins’) involves HC protein HSP90AB1 that is part of the heat-shock response. The prominent involvement of chaperone pathways alongside a heat-shock response is evidence that expression of FGFR3-TACC3 fusion alone promotes stress response pathways in this cell line, independent of other stress-promoting factors. The localised phospho-signalling context of LMNA, SSR1 and TLN1 is shown in Supplementary Figure 6. From this, one of the strongest C1-only phosphosites (TLN1 Y70) is predicted to be phosphorylated by FGR, which has a functional interaction with SHC1. Evidence supporting SHC1 involvement comes from interactions with proteins EGFR and EPHA2, both identified in the proteome dataset. LMNA shows reduced phosphorylation at S404/6, with the most likely kinase being GSK3A, which is also implicated in both AKT1 phosphosite up-regulation (S126/9) as well as up-regulation at S215 and down regulation at S219 of GSK3B.

**Figure 6:**
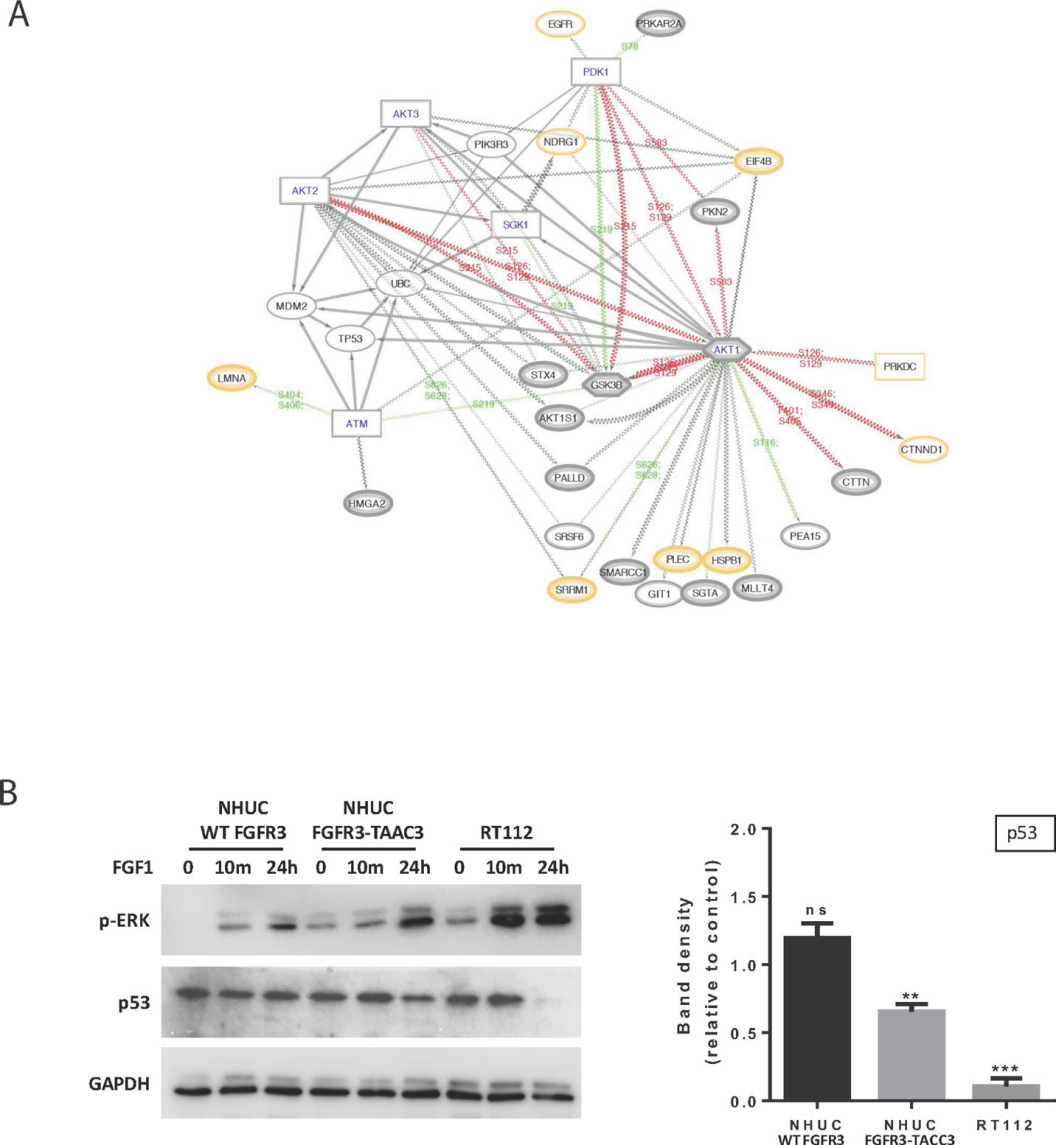
Further analysis of the relationship between FUS and TP53 based on C1 Reactome pathways 3 and 4 (**A**) Network diagram expanding of local functional interactions around pathways for the regulation of TP53 expression and degradation (C1-only, 3 & 4). Observed phosphoproteins (ovals) and predicted interacting kinases (rectangles; hexagons if also substrate) shown with significant up or down regulated phosphosite changes (red/green lines respectively). (QP proteins medium grey border; HC thick grey border; proteome yellow border). The diagram shows the central importance of AKT1 in FUS in terms of multiple predicted functional interactions, including TP53, EIF4B, GSK3B and PDK1. AKT1, 2 and 3 all have functional interactions with either TP53 or MDM2. The role of the predicted ATM kinase in these interactions is supported by links to substrates LMNA and HMGA2. (**B**) Western blotting (left panel) showing p-ERK and TP53 levels in NHUC cells stably expressing FGFR3 (IIIb) WT or FGFR3-TACC3 and the RT112 cell line following stimulation with 100 ng/ml FGF1 and 100 IU/ml Heparin for the indicated time points. GAPDH was used as a loading control. Quantification of TP53 levels after 24-hour stimulation compared to unstimulated cells (right panel) shows that TP53 is significantly down-regulated in the RT112 cells and in the NHUC FGFR3-TACC3 expressing cells, whereas no change is seen in NHUC cells expressing FGFR3 (IIIb) WT (*n* = 4 separate blots from 2 biological repeats, ± SEM). Data was analysed by multiple one-sample *t*-tests to a normalised control of 1 with ^**^P < 0.01 and ^***^P < 0.001.

We additionally analysed C1-only pathways 3 & 4, which form a pair of pathways relating to TP53 expression and degradation (Figure 5 and Figure 6A). Although TP53 and MDM2 are not observed directly (i.e. in HC, QP or proteome), we are able to infer their likely role in FUS signalling via our DRAGON protocol and provide a network of their interactions (based on data from Reactome FIViz [34]) including many proteins that are directly observed in our proteomics datasets; examples include AKT1, EIF4B and GSK3B (Figure 6A and Supplementary Table 1). Together, these networks and pathways imply that FUS signalling could act to reduce apoptosis via enhanced signalling of the AKT / mTOR pathways, a pro-survival outcome mediated via activation of MDM2 [35].

Shared proteins and pathways provide evidence for interplay between C1-only and C3-only processes (Figure 5). For example, although the pathway C1-only [7] - ‘Negative regulation of MET activity’ is supported by only one HC protein, HGS, this is one of the clearest up-regulated phosphosites in C1-only (Figure 3A) and this pathway connects (via GRB2) to the significantly fusion-regulated C1-C3-shared pathway [2] - ‘Spry regulation of FGF signalling’.

Considering the potential importance of the link between FGFR3-TACC3 and TP53 for the process of oncogenesis, we performed further experiments using NHUC cell lines (Figure 6B). We used FGF1 non-stimulated and stimulated conditions based on previous reports that FGF1 not only stimulates WT FGFR3 but also enhances signalling by FGFR3-TACC3 fusion [20]. We similarly observed higher p-ERK following stimulation in both NHUC cells expressing FGFR3-TACC3 and the original urothelial cancer cell line RT112 (Figure 6B, left panel). Quantitation of TP53 amounts under these conditions, in contrast, revealed a significant reduction, which was not detected in cells expressing WT FGFR3 (Figure 6B, right panel).

## DISCUSSION

FGFR-TACC fusions have emerged as one of the most significant chromosomal translocations in a range of cancer types [10–13]. One of the challenges related to a better understanding of the function of novel fusion kinase proteins has been to identify the signalling connectivity that is either modulated or completely “rewired” by them, since it is likely that these events are important in the transforming ability of the fusion kinase. We here address this challenge by focusing on FGFR3-TACC3 fusion in the context of a human urothelial cell line (TERT-NHUC) with particular relevance for development of bladder cancer.

The combination of powerful individual bioinformatics tools and databases into a novel analysis protocol, DRAGON, allows data from a large number of phosphosite quantifications to be interpreted in terms of the differentially affected pathways between two comparisons, without requiring prior assumptions as to those most likely affected. DRAGON uniquely expands and filters protein interaction networks in multiple steps (Figure 4 and Supplementary Experimental Procedures). DRAGON thus provides a succinct interpretation of phosphosite changes by using both local (kinase-substrate) and genome wide (biological pathway) signalling contexts. The final DIAMOnD-enriched lists of proteins for C1 (*n* = 354) and C3 (*n* = 347) and the networks of functional interactions (Figures 5, 6, Supplementary Figures 5, 6) are consistent with increasing evidence that cells are highly integrated complex systems in which functional changes may be reminiscent of phase changes and potentially involve hundreds of proteins [36, 37]. Proteome-wide data and careful filtering are needed to identify the most relevant protein/pathway networks and to eliminate unlikely and unhelpful predictions.

In the context of changes in phosphorylation, we found 9 pathways significantly regulated only in the cell line expressing FGFR3-TACC3 fusion, including 3 related to chaperone activation and stress response and 2 to regulation of TP53 expression and degradation. Of the 5 pathways most clearly significantly regulated only by stimulation of the WT FGFR3, 2 relate to apoptotic execution phase, with a further pathway governing TP53 regulation by acetylation. These mutually exclusive sets suggest that in addition to modulation of the nine common pathways identified in our experiments, there are also “re-wiring” differences between FUS and WT cells.

While the specific observed differences require further investigation, their potential significance can be further considered based on information from previous studies of bladder and other cancers and related cancer cell lines. With respect to unique pathways regulating chaperone activation and stress response (Figure 5), some of the pathways (ER stress response) have been well-established as a consequence of oncogene expression in general and a higher rate of protein synthesis; accumulating evidence indicates that this stress response contributes to the development of cancer, affecting diverse aspects of the disease [38, 39]. In contrast, the link with the HSP90 chaperone system, has been previously associated with a subset of proteins (clients) including oncogenic fusion kinases [40, 41] and specifically documented for FGFR3-TACC3 fusion in the context of a bladder cancer cell line, RT112 [42]. The enhanced dependence of the FGFR3-TACC3 fusion on the functional HSP90 has been documented by the loss of FGFR3-TACC3 fusion protein expression and depletion of multiple oncogenic signalling proteins in RT112 cells by a selective HSP90 inhibitor, resulting in potent cytotoxicity. Therefore, the increase in the abundance and/or functionality of the HSP90 system would provide considerable advantage to affected cells by maintaining overexpression levels of FGFR3-TACC3, associated with this chromosomal translocation. All of these observations support the conclusion that our methods are correctly identifying important functional differences.

Of significant interest are highlighted links between FGFR3-TACC3 and TP53 (Figures 5 and 6). Notably, the role of the master tumour suppressor TP53 in development of bladder cancer has been extensively studied with aberrations in this gene linked to more advanced and invasive stages of bladder cancer, i.e. cancer progression [18, 43]. In contrast, mutations in FGFR3 are present in both earlier and latter stages but with higher frequencies (about 80%) at early stages of cancer initiation and are regarded as a distinct path in urothelial tumorigenesis. Our phosphoproteomic data, supported by follow up functional studies, demonstrate for the first time that the FGFR3-TACC3 fusion protein is able to down-regulate TP53, both in the TERT-NHUC cell line and in the fully transformed RT112 cell line (Figure 6). Although not noted as a common effect, there are examples of a link between oncogene action and down-regulation of the wild-type TP53; among others, they include overexpression of HER-2/neu in breast cancer cell lines, NPM-ALK fusion protein in ALK-expressing lymphoma, cancer associated fibroblasts, and interestingly, another FGFR3-fusion protein, FGFR3–BAIAP2L1, where evidence has been based on a comprehensive gene expression analysis [35, 44–46]. Like FGFR3-TACC3, FGFR3–BAIAP2L1 has been identified in different cancers, including bladder cancer clinical samples and a bladder cancer cell line (SW780) [9, 44]. Both RT112 and SW780 urothelial cancer cell lines have the WT TP53; analysis of GMB clinical samples expressing FGFR3-TACC3, similarly, did not report correlation with mutations in TP53 [14]. It is possible that this FGFR3-fusion signalling to down regulate functions of TP53 in the context of urothelial cells contributes to changes towards the transformed phenotype even in the absence of TP53 mutations, initially by maintaining pre-malignant phenotype and allowing for accumulation of additional genetic alterations.

FGFR-TACC fusions are not limited to bladder cancer and have been observed in a range of different cancer types suggesting that some of the key differences could be common regardless of the tumour origin [10–13]. However, future global studies covering different cell types are needed to identify such key, common alterations. Similarly, the higher sensitivity to FGFR-inhibitors observed in FGFR3-fusion expressing cancer cells, compared to FGFR3 point mutations [19], could be better understood by comprehensive analyses and comparison of cellular networks linked to these different FGFR3 aberrations.

Overall, the tools used here offer a powerful approach for generating insights into cellular processes dysregulated by the fusion that contribute to subsequent transformation or maintenance of the cancer phenotype, thus providing a route to suggest additional or alternative therapeutic targets and potential biomarkers. While a number of the proteins/pathways/networks we identified have previously been individually associated with cancer in various cell types and contexts, the present results provide evidence of integrated interactions between these networks in a single system. Phosphoproteomics has thus provided an attractive initial overview of the complexity, but unravelling phase changes in highly integrated complex systems will require additional data and cell types. The DRAGON approach is not limited to phosphorylation networks and can potentially be applied in other analytical contexts of genomics, such as mRNA transcription, or proteomics, including regulation of protein abundance by degradation or translational control, other post-translation modifications and subcellular spatial modulation of function.

## MATERIALS AND METHODS

### Cell culture and FGF1 stimulation

Telomerase immortalised normal human urothelial cells (TERT-NHUC) expressing wild-type FGFR3 (IIIb) or FGFR3-TACC3 were generated previously [9] and cultured using the Keratinocyte Growth Medium Kit 2 (Promocell, Heidelberg, Germany). The RT112 bladder cell line was cultured in RPMI supplemented with 10% foetal bovine serum (Thermo Fisher Scientific, UK). Cells were maintained at 37°C, 5% CO2.

For FGF1 stimulation, cells were serum/supplement starved for 1 hour before incubation with 100 IU/ml heparin (Sigma-Aldrich, GIllingham, UK) with or without 100 ng/ml FGF1 (R&D Systems) for the indicated time points.

### Protein extraction, trypsinization and labelling

For proteomic experiments, protein lysates were obtained using RIPA buffer (Thermo Fisher Scientific, UK) supplemented with protease inhibitory cocktail (Sigma-Aldrich, Gillingham, UK) and 0.3 mM Na3VO4. Protein concentration was determined with the Pierce™ BCA Protein Assay Kit (Thermo Fisher Scientific, UK).

Samples were diluted to 1 mg/ml in 0.1 M TEAB, reduced with 10 mM DTT at 60°C, and alkylated with 25 mM IAA. Trypsinization was carried out for 16 hours at 37°C using Trypsin-Gold (Promega, Southampton, UK). Peptides were desalted using the Sep-Pak C18 cartridges (Waters, Elmstree, UK) before peptide labelling with the TMTsixplex™ Isobaric Label Reagent (Thermo Fisher Scientific, UK) kit according to the manufacturer’s instructions. Additional information can be found in the Supplementary Materials and Methods.

### Phosphopeptide enrichment workflows

Enrichment of the fraction of phosphorylated peptides utilised antibody precipitation coupled with TiO2 chromatography as described previously [26], with minor changes. For the anti-phosphotytrosine peptide immunoprecipitation, the antibody PT66 was replaced with pY1000 (Cell Signalling Technology, Hitchin, Hertfordshire, UK). Elution was performed with 0.5% formic acid, negating the need for an additional clean-up step. Additional information can be found in the Supplementary Materials and Methods.

### LC MS-MS and proteomic/phosphoproteomic data identification and quantification

LC-MS/MS analysis was performed using an LTQ-Velos Orbitrap mass spectrometer (Thermo Fisher Scientific) as described previously [26, 47]. A detailed description of data analysis can be found in the Supplementary Materials and Methods.

### Immunoprecipitation and blotting

Immunoblotting was carried out using 15 or 30 µg of total protein lysate as described previously. The following primary antibodies were used: anti-β-actin and anti-Laminin (Abcam, Cambridge, UK); anti-ERK1 K-23 and anti-FGFR3 B-9 (Santa Cruz Biotechnology); anti-Phospho ERK1/2, T202/Y204, anti-α-E-Catenin 23B2 and anti-Phospho-α-E-Catenin S652 (Cell Signalling, Hitchin, Hertfordshire, UK). Anti-mouse or anti-rabbit IgG HRP linked secondary antibodies (Cell Signalling, Hitchin, Hertfordshire, UK) were used.

The PathScan^®^ Intracellular Signalling Array Kit (Cell Signalling, Hitchin, Hertfordshire, UK) was used according to manufacturer’s instructions to explore the phosphorylation status of proteins fundamental to signal transduction. Slide images were captured using Odyssey Fc System (Li-Cor Biosciences).

Immunoprecipitation for both FGFR3 WT and FGFR3-TACC3 (RT112) fusion were performed as in [48]. Immunoprecipitation of Topoisomerase IIα used 100 ug of starting material and was precipitated using the anti-TOPO IIα antibody (Cell Signalling, Hitchin, Hertfordshire, UK). Eluted protein was probed via immunoblotting using TOPO IIα and pS 1469 TOPO IIα antibodies (Cell Signaling, Hitchin, Hertfordshire, UK). Additional information can be found in the Supplementary Materials and Methods.

### Differential pathways analysis by DRAGON protocol

Proteins identified from peptides containing significantly altered phosphosites (defined as those sites where the quantitation ratio varied by at least 1.96σ with respect to WT) were mapped to kinases using NetworKIN [29, 49]. NetworKIN calculates an overall likelihood ratio that an observed phosphosite was phosphorylated by a particular kinase by combining individual likelihood ratios derived from local network proximity (*via* STRING [50] network topology) and the probability of interaction at the phosphosite’s peptide sequence motif, obtained via NetPhorest [51] classifiers. To remove the least likely kinases for each site, kinases with likelihoods below the median NetworKIN score (∼1) were filtered out. An effective network was modelled for comparisons C1 and C3 by extracting a sub-network containing the combined set of QP identified proteins and their predicted interacting kinase or SH2-domain containing proteins (together forming the protein “seeds”), and their network neighbours from a comprehensive human signalling network compiled from Pathway Commons [52]. Each resulting effective network was filtered using Human Protein Atlas [53] expression levels to remove proteins unlikely to be expressed in either urothelial cancer or normal urothelial cells. The seed set was expanded by finding a further 200 disease-related proteins for each condition by applying the DIAMOnD [31] algorithm to identify proteins showing significant connectivity with seeds on the HSN. For each condition in turn, Reactome [28, 54] pathway enrichment was tested using the combined set of seeds and significantly connected proteins, discarding pathways that did not contain at least one HC protein. The resulting pathway lists were compared to identify those unique to one condition or the other - the “differential pathways”. Additionally, pathways common to both conditions that contained 25% more protein hits in either one were marked as “differentially regulated shared pathways”. Detailed information can be found in the Supplementary Materials and Methods.

## SUPPLEMENTARY MATERIALS FIGURES AND TABLES













## References

[1] Belov AA, Mohammadi M (2013). Molecular mechanisms of fibroblast growth factor signaling in physiology and pathology. Cold Spring Harb Perspect Biol.

[2] Helsten T, Schwaederle M, Kurzrock R (2015). Fibroblast growth factor receptor signaling in hereditary and neoplastic disease: biologic and clinical implications. Cancer Metastasis Rev.

[3] Itoh N, Ornitz DM (2011). Fibroblast growth factors: from molecular evolution to roles in development, metabolism and disease. J Biochem.

[4] Touat M, Ileana E, Postel-Vinay S, Andre F, Soria JC (2015). Targeting FGFR Signaling in Cancer. Clin Cancer Res.

[5] Katoh M (2016). Therapeutics Targeting FGF Signaling Network in Human Diseases. Trends Pharmacol Sci.

[6] Carter EP, Fearon AE, Grose RP (2015). Careless talk costs lives: fibroblast growth factor receptor signalling and the consequences of pathway malfunction. Trends Cell Biol.

[7] Gallo LH, Nelson KN, Meyer AN, Donoghue DJ (2015). Functions of Fibroblast Growth Factor Receptors in cancer defined by novel translocations and mutations. Cytokine Growth Factor Rev.

[8] Singh D, Chan JM, Zoppoli P, Niola F, Sullivan R, Castano A, Liu EM, Reichel J, Porrati P, Pellegatta S, Qiu K, Gao Z, Ceccarelli M (2012). Transforming fusions of FGFR and TACC genes in human glioblastoma. Science.

[9] Williams SV, Hurst CD, Knowles MA (2013). Oncogenic FGFR3 gene fusions in bladder cancer. Hum Mol Genet.

[10] Parker BC, Engels M, Annala M, Zhang W (2014). Emergence of FGFR family gene fusions as therapeutic targets in a wide spectrum of solid tumours. J Pathol.

[11] Sabnis AJ, Bivona TG (2013). FGFR fusions in the driver's seat. Cancer Discov.

[12] Costa R, Carneiro BA, Taxter T, Tavora FA, Kalyan A, Pai SA, Chae YK, Giles FJ (2016). FGFR3-TACC3 fusion in solid tumors: mini review. Oncotarget.

[13] Lasorella A, Sanson M, Iavarone A (2017). FGFR-TACC gene fusions in human glioma. Neuro Oncol.

[14] Di Stefano AL, Fucci A, Frattini V, Labussiere M, Mokhtari K, Zoppoli P, Marie Y, Bruno A, Boisselier B, Giry M, Savatovsky J, Touat M, Belaid H (2015). Detection, Characterization, and Inhibition of FGFR-TACC Fusions in IDH Wild-type Glioma. Clin Cancer Res.

[15] Tabernero J, Bahleda R, Dienstmann R, Infante JR, Mita A, Italiano A, Calvo E, Moreno V, Adamo B, Gazzah A, Zhong B, Platero SJ, Smit JW (2015). Phase I Dose-Escalation Study of JNJ-42756493, an Oral Pan-Fibroblast Growth Factor Receptor Inhibitor, in Patients With Advanced Solid Tumors. J Clin Oncol.

[16] Gergely F, Kidd D, Jeffers K, Wakefield JG, Raff JW (2000). D-TACC: a novel centrosomal protein required for normal spindle function in the early Drosophila embryo. EMBO J.

[17] Wu YM, Su F, Kalyana-Sundaram S, Khazanov N, Ateeq B, Cao X, Lonigro RJ, Vats P, Wang R, Lin SF, Cheng AJ, Kunju LP, Siddiqui J (2013). Identification of targetable FGFR gene fusions in diverse cancers. Cancer Discov.

[18] Knowles MA, Hurst CD (2015). Molecular biology of bladder cancer: new insights into pathogenesis and clinical diversity. Nat Rev Cancer.

[19] di Martino E, Tomlinson DC, Williams SV, Knowles MA (2016). A place for precision medicine in bladder cancer: targeting the FGFRs. Future Oncol.

[20] Parker BC, Annala MJ, Cogdell DE, Granberg KJ, Sun Y, Ji P, Li X, Gumin J, Zheng H, Hu L, Yli-Harja O, Haapasalo H, Visakorpi T (2013). The tumorigenic FGFR3-TACC3 gene fusion escapes miR-99a regulation in glioblastoma. J Clin Invest.

[21] Yuan L, Liu ZH, Lin ZR, Xu LH, Zhong Q, Zeng MS (2014). Recurrent FGFR3-TACC3 fusion gene in nasopharyngeal carcinoma. Cancer Biol Ther.

[22] Carneiro BA, Elvin JA, Kamath SD, Ali SM, Paintal AS, Restrepo A, Berry E, Giles FJ, Johnson ML (2015). FGFR3-TACC3: A novel gene fusion in cervical cancer. Gynecol Oncol Rep.

[23] Daly C, Castanaro C, Zhang W, Zhang Q, Wei Y, Ni M, Young TM, Zhang L, Burova E, Thurston G (2017). FGFR3-TACC3 fusion proteins act as naturally occurring drivers of tumor resistance by functionally substituting for EGFR/ERK signaling. Oncogene.

[24] Nelson KN, Meyer AN, Siari A, Campos AR, Motamedchaboki K, Donoghue DJ (2016). Oncogenic Gene Fusion FGFR3-TACC3 Is Regulated by Tyrosine Phosphorylation. Mol Cancer Res.

[25] von Stechow L, Francavilla C, Olsen JV (2015). Recent findings and technological advances in phosphoproteomics for cells and tissues. Expert Rev Proteomics.

[26] Lombardi B, Rendell N, Edwards M, Katan M, Zimmermann JG (2015). Evaluation of phosphopeptide enrichment strategies for quantitative TMT analysis of complex network dynamics in cancer-associated cell signalling. EuPA Open Proteom.

[27] Petsalaki E, Helbig AO, Gopal A, Pasculescu A, Roth FP, Pawson T (2015). SELPHI: correlation-based identification of kinase-associated networks from global phospho-proteomics data sets. Nucleic Acids Res.

[28] Fabregat A, Sidiropoulos K, Garapati P, Gillespie M, Hausmann K, Haw R, Jassal B, Jupe S, Korninger F, McKay S, Matthews L, May B, Milacic M (2016). The Reactome pathway Knowledgebase. Nucleic Acids Res.

[29] Horn H, Schoof EM, Kim J, Robin X, Miller ML, Diella F, Palma A, Cesareni G, Jensen LJ, Linding R (2014). KinomeXplorer: an integrated platform for kinome biology studies. Nat Methods.

[30] Cox J, Mann M (2008). MaxQuant enables high peptide identification rates, individualized p.p.b.-range mass accuracies and proteome-wide protein quantification. Nat Biotechnol.

[31] Ghiassian SD, Menche J, Barabasi AL (2015). A DIseAse MOdule Detection (DIAMOnD) algorithm derived from a systematic analysis of connectivity patterns of disease proteins in the human interactome. PLoS Comput Biol.

[32] Sharma A, Menche J, Huang CC, Ort T, Zhou X, Kitsak M, Sahni N, Thibault D, Voung L, Guo F, Ghiassian SD, Gulbahce N, Baribaud F (2015). A disease module in the interactome explains disease heterogeneity, drug response and captures novel pathways and genes in asthma. Hum Mol Genet.

[33] Arakawa H (2004). Netrin-1 and its receptors in tumorigenesis. Nat Rev Cancer.

[34] Wu G, Dawson E, Duong A, Haw R, Stein L (2014). ReactomeFIViz: a Cytoscape app for pathway and network-based data analysis. F1000Res.

[35] Zheng L, Ren JQ, Li H, Kong ZL, Zhu HG (2004). Downregulation of wild-type p53 protein by HER-2/neu mediated PI3K pathway activation in human breast cancer cells: its effect on cell proliferation and implication for therapy. Cell Res.

[36] Pinto G, Radulovic M, Godovac-Zimmermann J (2016 May 17). Spatial perspectives in the redox code-Mass spectrometric proteomics studies of moonlighting proteins. Mass Spectrom Rev.

[37] Radulovic M, Baqader NO, Stoeber K, Godovac-Zimmermann J (2016). Spatial Cross-Talk between Oxidative Stress and DNA Replication in Human Fibroblasts. J Proteome Res.

[38] Hetz C, Chevet E, Harding HP (2013). Targeting the unfolded protein response in disease. Nat Rev Drug Discov.

[39] Dufey E, Urra H, Hetz C (2015). ER proteostasis addiction in cancer biology: Novel concepts. Semin Cancer Biol.

[40] Taipale M, Jarosz DF, Lindquist S (2010). HSP90 at the hub of protein homeostasis: emerging mechanistic insights. Nat Rev Mol Cell Biol.

[41] Calderwood SK, Gong J (2016). Heat Shock Proteins Promote Cancer: It’s a Protection Racket. Trends Biochem Sci.

[42] Acquaviva J, He S, Zhang C, Jimenez JP, Nagai M, Sang J, Sequeira M, Smith DL, Ogawa LS, Inoue T, Tatsuta N, Knowles MA, Bates RC, Proia DA (2014). FGFR3 translocations in bladder cancer: differential sensitivity to HSP90 inhibition based on drug metabolism. Mol Cancer Res.

[43] Wu XR (2005). Urothelial tumorigenesis: a tale of divergent pathways. Nat Rev Cancer.

[44] Nakanishi Y, Akiyama N, Tsukaguchi T, Fujii T, Satoh Y, Ishii N, Aoki M (2015). Mechanism of Oncogenic Signal Activation by the Novel Fusion Kinase FGFR3-BAIAP2L1. Mol Cancer Ther.

[45] Cui YX, Kerby A, McDuff FK, Ye H, Turner SD (2009). NPM-ALK inhibits the p53 tumor suppressor pathway in an MDM2 and JNK-dependent manner. Blood.

[46] Procopio MG, Laszlo C, Al Labban D, Kim DE, Bordignon P, Jo SH, Goruppi S, Menietti E, Ostano P, Ala U, Provero P, Hoetzenecker W, Neel V (2015). Combined CSL and p53 downregulation promotes cancer-associated fibroblast activation. Nat Cell Biol.

[47] Pinto G, Alhaiek AA, Amadi S, Qattan AT, Crawford M, Radulovic M, Godovac-Zimmermann J (2014). Systematic nucleo-cytoplasmic trafficking of proteins following exposure of MCF7 breast cancer cells to estradiol. J Proteome Res.

[48] di Martino E, LʼHote CG, Kennedy W, Tomlinson DC, Knowles MA (2009). Mutant fibroblast growth factor receptor 3 induces intracellular signaling and cellular transformation in a cell type- and mutation-specific manner. Oncogene.

[49] Linding R, Jensen LJ, Ostheimer GJ, van Vugt MA, Jorgensen C, Miron IM, Diella F, Colwill K, Taylor L, Elder K, Metalnikov P, Nguyen V, Pasculescu A (2007). Systematic discovery of *in vivo* phosphorylation networks. Cell.

[50] Franceschini A, Szklarczyk D, Frankild S, Kuhn M, Simonovic M, Roth A, Lin J, Minguez P, Bork P, von Mering C, Jensen LJ (2013). STRING v9.1: protein-protein interaction networks, with increased coverage and integration. Nucleic Acids Res.

[51] Miller ML, Jensen LJ, Diella F, Jorgensen C, Tinti M, Li L, Hsiung M, Parker SA, Bordeaux J, Sicheritz-Ponten T, Olhovsky M, Pasculescu A, Alexander J (2008). Linear motif atlas for phosphorylation-dependent signaling. Sci Signal.

[52] Cerami EG, Gross BE, Demir E, Rodchenkov I, Babur O, Anwar N, Schultz N, Bader GD, Sander C (2011). Pathway Commons, a web resource for biological pathway data. Nucleic Acids Res.

[53] Uhlen M, Fagerberg L, Hallstrom BM, Lindskog C, Oksvold P, Mardinoglu A, Sivertsson A, Kampf C, Sjostedt E, Asplund A, Olsson I, Edlund K, Lundberg E (2015). Proteomics. Tissue-based map of the human proteome. Science.

[54] Croft D, Mundo AF, Haw R, Milacic M, Weiser J, Wu G, Caudy M, Garapati P, Gillespie M, Kamdar MR, Jassal B, Jupe S, Matthews L (2014). The Reactome pathway knowledgebase. Nucleic Acids Res.

