# Supplementary material for: Unique signalling connectivity of FGFR3-TACC3 oncoprotein revealed by quantitative phosphoproteomics and differential network analysis
Source: Oncotarget. 2017 Oct 25;8(61):102898–911. doi: 10.18632/oncotarget.22048 (PMC5732698; doi:10.18632/oncotarget.22048)
Supplement: Supplementary file 1 [file oncotarget-08-102898-s001.pdf]

Unique signalling connectivity of FGFR3-TACC3 oncoprotein revealed by quantitative phosphoproteomics and differential network analysis

SUPPLEMENTARY MATERIALS

SUPPLEMENTARY INFORMATION

Supplementary Experimental Procedures

Analysis of cell lines and proteomic methods

Extended methods for cell lysis, protein extraction, trypsinization and labelling

For proteomic experiments, protein lysates were obtained by adding RIPA buffer (Thermo Fisher Scientific, UK) supplemented with protease inhibitory cocktail (Sigma-Aldrich, Gillingham, UK) and 0.3 mM Na_3VO_4 directly on the cell layer, previously washed twice with cold PBS. The lysates were kept on ice for 15 minutes, occasionally vortexed and then clarified by centrifugation at 16.000 x *g* for 15 min at 4°C. The resulting supernatant was aliquoted and kept at S –80°C for subsequent processing steps. Protein concentration was determined with the Pierce™ BCA Protein Assay Kit (Thermo Fisher Scientific, UK) and 3 replicates of 0.5 mg samples were used for analysis of each condition/experiment.

Samples were diluted in 0.1 M TEAB (Thermo Fisher Scientific, UK) to a concentration of 1 mg/ml, reduced with 10 mM DTT (Sigma-Aldrich, Poole, UK) for 10 min at 60°C and then alkylated with 25 mM IAA (Sigma-Aldrich, Poole, UK) for 30 min at room temperature, protected from light. Trypsin-Gold (Promega, Southampton, UK) was added in 1:50 ratio (enzyme : total lysate) and incubated for 16 hours at 37°C. Peptides were desalted using the Sep-Pak C18 cartridges (Waters, Elmstree, UK) and eluted with 25% acetonitrile (Sigma-Aldrich, Poole, UK). Peptide labelling with the TMTsixplex™ Isobaric Label Reagent (Thermo Fisher Scientific, UK) kit was performed according to the manufacturers instructions, with each sample/replicate mixed with 2 vials (1.6 mg) of labelling reagent. Samples were gently agitated at room temperature for 1 hour, then quenched with the addition of hydroxylamine (Thermo Fisher Scientific, UK). An aliquot of each sample was checked for labelling efficiency >98% before proceeding with the further phosphopeptide enrichment workflow. Moreover, an aliquot (TMT mixture) containing all samples mixed in nominally equal proportions was used to check and eventually correct sample loading imbalances before proceeding with the phosphopeptide enrichment steps.

Extended methods for phosphopeptide enrichment

The labelled peptides isolated from the four samples were mixed in equal proportions and the workflow undertaken to enrich the fraction of phosphorylated peptides from the unphosphorylated ones is based on the "workflow 2" previously described [1] with minor changes. Briefly, for the anti-phosphotytrosine peptide immunoprecipitation, the antibody PT66 (Sigma-Aldrich, Poole, UK) was replaced with pY1000 (Cell Signaling Technology, Hitchin, Hertfordshire, UK), while 4G10 (Millipore) and pY100 (Cell Signaling Technology, Hitchin, Hertfordshire, UK) were kept. Elution was performed with 0.5% formic acid so that the sample could be directly injected into the mass spectrometer without the need for an additional clean-up step.

The TiO_2 chromatography was performed on the unbound fraction using the TiO_2 phosphopeptide enrichment kit (Thermo Fisher Scientific, UK) followed by a clean-up step with graphite columns (Thermo Fisher Scientific, UK), according to manufacturer's instructions, and samples were prepared for injection as previously described [1]. The flow through (FT) collected after the TiO_2 chromatographic step was kept, with an aliquot corresponding to 1/50 of the available volume desalted with the Zeba™ Spin Desalting Column (Thermo Fisher Scientific, UK) injected into the mass spectrometer for normalisation purposes and overall proteomic evaluation of the samples.

Extended methods for LC MS-MS and proteomic/phosphoproteomic data identification and quantification

LC-MS/MS analysis was performed using an LTQ-Velos Orbitrap mass spectrometer (Thermo Fisher Scientific, UK). Peptide samples were loaded using nanoACQUITY UPLC (Waters, Elstree, U.K.) with Symmetry C18 180 µm × 20 mm (Waters, Elstree, UK, part number 186006527) trapping column for desalting and then introduced into the mass spectrometer via Stonearch fused silica capillary column: 100 µm i.d., 360 µm o.d., 15 cm length, 5 µm C18 particles (Nikkyo Technos CO, Tokyo, Japan part number NTCC 360/100-5-153) and a nanoelectrospray ion source at a flow rate of

0.40 μL/min. The mobile phase comprised H_2O with 0.1% formic acid (buffer A) and 100% acetonitrile with 0.1% formic acid (buffer B). The gradient ranged from 1% to 50% buffer B in 136 min and a step gradient to 85% B for 20 min with a flow of 0.40 μL/min, finally a return to the initial conditions of 1% B for 20 min [1, 2].

The full scan precursor survey MS spectra (400–2000 m/z) were acquired in full profile with the Velos-Orbitrap analyser at a resolution of r = 60,000.

This was followed by data dependent MS/MS fragmentation in centroid mode of the most intense ion from the survey scan: electrospray voltage 1.5 kV, capillary temperature 200°C and isolation width 2.00. First, using collision induced dissociation (CID) in the linear ion trap: normalized collision energy 35%, activation Q 0.25 and activation time 10 ms. For the second MS/MS event, the most intense ion from the survey scan was fragmented using higher energy collision dissociation (HCD) in the HCD collision cell: normalized collision energy 50%, resolution 7500 and activation time 0.1 ms. The two MS/MS scan events were repeated for the top 10 peaks in the MS survey scan, the targeted ions were then dynamically excluded for 30 s. Singly charged ions were excluded from the MS/MS analysis and Xcalibur software version 2.1.0 SP1 build 1160 (Thermo Fisher Scientific, U.K.) used for data acquisition. Raw data were analysed using Proteome Discoverer (PD v1.3) with Mascot search engine and Swiss-Prot human proteome database (v11/16). Up to 2 trypsin missed cleavages were allowed, carbamidomethylation was set as a fixed modification, while methionine oxidation, phosphorylation of serine, threonine and tyrosine were set as variable modifications. Furthermore, TMT 6-Plex labelling was set as variable modification to evaluate the labelling efficiency of each sample, but set as fixed during the analysis of phosphopeptide-enriched samples and of the unbound fraction. Mass tolerance was set to 7 ppm for the precursors and to 0.8 Da for the fragments. FDRs were chosen at the peptide level to generate two different datasets: High Confidence Quantitative Phosphoproteomic dataset (HC) with an FDR $\leq$ 0.01, and Quantitative Phosphoproteomic dataset (QP) with FDR $\leq$ 0.05.

In order to identify and quantify phosphopeptides, a workflow was created in PD to sequence a peptide based on CID-derived spectra and assign a quantification based on the corresponding HCD-derived spectra. To statistically evaluate peptide identifications and obtain probabilistic scores for phosphorylation site(s) localisation, both the Peptide validator and the phosphoRS [3] nodes were added to the workflow. Phosphosite localisation was manually inspected if the PhosphoRS probability was lower than 0.75. The quantitative information obtained from analysis of the FT was used to normalise the samples and calculate a correction factor to account for systematic bias incorporated in peptide measurements - for example, due to variations in sample loading.

A total intensity normalisation approach was applied, which is based on the assumption that the total intensity of each reporter ion for each sample remains the same in the four conditions [4]. The correction factors, calculated from the unbound fractions, were used to normalise the intensities of the reporter ions collected in the phosphopeptide-enriched fractions (IP and TiO_2).

Phosphosite localisation was finalised by choosing the assignment corresponding to the highest probability score calculated by PhosphoRS. Phosphopeptides having the same phosphorylation pattern across one or more replicates were grouped together and relative ratios (FUS Hep: WT Hep; FUS FGF1: WT FGF1; WT FGF1: WT Hep) were obtained by averaging the quantitative information obtained from each data point in each replicate. Finally, a mean ratio was obtained, averaging the ratios obtained in each individual replicate, where available. Standard deviations and coefficients of variation were calculated when more than one data point (*i.e.* measurement per replicate) was available.

Statistical evaluation of differentially changing phosphosites (including SigB significance) in each condition was calculated using Perseus v.1.5.5.3.

The reference proteome was obtained by analysing the sample prior to enrichment (TMT mix) and TiO_2 FT. The FDR at the peptide level was set at 0.01 and was required to have at least 1 unique quantified peptide per protein to include the identified protein into the dataset. For quantification, the intensities were normalised over the overall protein median, as PD workflow suggests in its settings.

Extended methods for Immunoprecipitation and blotting

15 or 30 μg of total protein lysates were loaded in a 4–15% or 7.5% (to visualize FGFR3 only) SDS polyacrylamide gel (Bio-rad, Watford, UK) and run under denaturing conditions. At the end of the run, separated proteins were transferred onto a nitrocellulose membrane using a semi-dry transfer system (Bio-rad, Watford, UK) for 120 minutes at 20 V. Membranes were blocked for 1 hour, under orbital agitation, either with 3% (w/v) NFDM (Bio-Rad) or 3% (w/v) BSA (Sigma, Aldrich, Poole, UK), depending on primary antibody requirements. Primary antibody incubations were performed overnight under orbital agitation at 4°C by diluting the antibodies accordingly to manufacturer's instructions; secondary antibodies were incubated for 1 hour under orbital agitation at room temperature. Immunoreactive bands were detected using the SuperSignal West Pico Chemiluminescent Substrate (Thermo Fisher Scientific, UK) and developed either by exposing the membrane to an X-ray film (Amersham) or by digital imaging. Densitometric analysis used ImageStudioLite (v. 5.2.5) for experiments run at least in triplicates on three independent biological replicates, while statistical evaluation of the results and graphing used Prism 7.

The PathScan® Intracellular Signaling Array Kit (Cell Signaling, Hitchin, Hertfordshire, UK) was used to explore the phosphorylation status of proteins fundamental to signal transduction. The assay was performed according to the manufacturer's instructions, with total lysates prepared in the lysis buffer provided in the kit and diluted to 0.35 mg/mL prior to incubation on the slides.

Slide images were captured using Odyssey Fc System (Li-Cor Biosciences) and analysed with ImageStudioLite v.5.2.5. The experiment was performed on total protein lysates from two independent growths, with densitometric analysis performed using ImageStudioLite. Signals were normalised towards the positive control, followed by the relative comparisons across all four conditions. Graphing and statistical analyses used Prism 7, unpaired Student's *t*-test and *p*-value cut-off = 0.05.

Immunoprecipitation for both FGFR3 and RT112FUS were performed as in [5]. Immunoprecipitation of Topoisomerase IIα used 100 ug of starting material. Total lysates and the anti-TOPO IIα antibody (Cell Signaling, Hitchin, Hertfordshire, UK) were mixed in 100:1 ratio (total lysate : antibody) and incubated over night at 4°C with gentle agitation. Protein A sepharose beads (Invitrogen, Thermo Fisher Scientific, UK) were added to the mixture and incubated for three hours under slow agitation. After a brief, gentle centrifugation (1 minute at 2000 g), the unbound fraction was removed, beads washed 5 times with lysis buffer (RIPA) and eluted by boiling the sample for 5 minutes in 3X Lamely sample buffer containing the reducing agent. Eluted samples were divided into two aliquots of equal volumes, with western blots to assess levels of expression of TOPO IIα and pS 1469 TOPO IIα (detected with the Cell Signaling, Hitchin, Hertfordshire, UK antibody).

Computational methods

Effective networks for C1 and C3

We modelled the signalling context of the kinase and substrate seed proteins relevant to each of the conditions C1 and C3 by creating a comprehensive human signalling network (HSN) from the pathway information collected in the Pathway Commons database [6]. All human proteins and their regulatory and physical interactions were included in the human signalling network with special attention to protein phosphorylation - we considered the Pathway Commons binary relations: "controls-expression-of", "interacts-with", "controls-phosphorylation-of", "controls-state-change-of" and "in-complex-with" as explained in www.pathwaycommons.org/pc2/formats.

We derived an effective network from the HSN for a set of 527 seed proteins (comprising all distinct proteins in the QP dataset and all those predicted to interact with any of the QP dataset phospho-sites according to the NetworKIN database). This effective network is a subnetwork of the HSN that contains the seed proteins and those directly affected by them, which are identified by applying a commute-time kernel (CT kernel) on the HSN.

The CT kernel matrix of the HSN integrates the network topology on the distance measures between nodes. Therefore, the CT-kernel provides a richer measure of the network distance indicating how effective the connection is between any two nodes in the HSN [7]. We mapped protein seeds onto the CT kernel and gradually incorporated proteins effectively connected to the seeds. To define the threshold of the CT kernel at which two proteins are considered effectively connected, we used the articulation nodes. The articulation nodes of a connected network are the minimum set of nodes that need to be removed in order to make it disconnected. In terms of 'information flow', any two nodes on a connected network will have a path connecting them, and any information flowing between them can be disrupted by removing a (relatively small) subset of nodes on the path; these articulation nodes are the essential elements in keeping the network connected and maintaining the integrity of information flow through it [8].

Starting from the 527 protein seeds, more network neighbours were gradually included by relaxing the threshold of the CT kernel-based distance. For each of these CT-kernel cut-offs, a number of network neighbours that are articulation nodes, plus other neighbours that are not essential to the network's integrity were included. A CT kernel cut-off that maximises the ratio of articulation nodes to total nodes was used to define the effective network, as shown in Illustration 1.

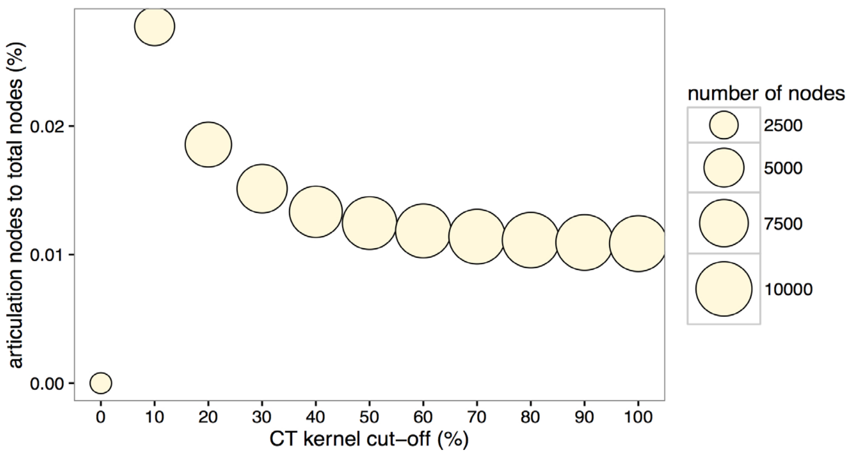

Illustration 1. Choice of cut-off for the CT kernel. The ratio of articulation to total nodes for each CT cut-off indicates the optimal choice in order to maximise the number of important articulation nodes, while minimising non-essential nodes.

From the overall CT kernel HSN containing 19,446 proteins and 755,299 interactions, the final effective network obtained using the 527 seeds and CT cut-off 10% contained 4,991 proteins and 308,564 edges. Further filtering was applied to the effective network to remove proteins not found in either urothelial cells or urothelial tumour cells (using Human Protein Atlas data - method described below) resulting in a final connected filtered effective network comprising 4,716 proteins and 281,403 interactions.

Generating detailed local networks

We identified Reactome pathways that were significantly differentially modulated when comparing FGFR3-TACC3 fusion cell signalling (i.e. condition C1) with FGF-induced signalling in FGFR3b-expressing cells (i.e. the wild-type model, condition C3). Detailed local networks were created by overlaying multiple sources of network information, including the Reactome database and a published functional interactome (Wu *et al* 2010) alongside other regulatory and physical interactions annotated in the effective network. Each of the detailed network figures (Supplementary Figures 5 and 6) was generated by expansion around the associated differential pathway proteins as outlined below.

Create an induced subgraph on the filtered effective network using the set of "seed" proteins connected to the relevant differential Reactome pathways, plus the proteins not found in Reactome during differential network analyses (as these are still part of the effective network) (Illustration 2).

Extend the network using the ReactomeFIViz tool [9]. This Cytoscape plugin uses a set of curated functional protein-protein interactions and a supplied list of proteins (here, all proteins in [1]). Proteins that link seeds to the non-Reactome proteins were found using the plugin's 'get linkers' option, alongside functional annotation of the interactions (Illustration 3).

Add predicted substrate-kinase and substrate-SH2 interactions from both the QP data and NetworKIN. All QP phospopeptide proteins and the predicted kinase/SH2 interactors were added to network [2] where either the substrate or the kinase is a member of network [2].

Filter interactions. The resulting network [3] was simplified by removing redundant or non-informative interactions where there are multiple edges between two proteins. Further filtering removed proteins that were weakly connected - i.e. if they had degree one and were not connected to any QP, HC or proteome proteins.

Annotate network at protein or interaction-level to indicate important features, e.g. by representing QP or HC proteins with bold outline, or by highlighting phospho-site interactions that significantly differ in their quantitation ratios between this condition (e.g. C3) and its comparator (i.e. C1) (Supplementary Figures 5 and 6).

Detailed networks generated for two differential pathways are presented in Supplementary Figures 5 and 6.

HPA processing

We obtained data from Human Protein Atlas (HPA) for normal tissue (downloaded 10th June 2016) and tumour (downloaded 17th June 2016). Protein expression levels for normal tissue data were used to filter out proteins from C3-based networks, by first filtering the HPA normal dataset by tissue/cell-type (tissue="urinary bladder" and cell type="urothelial cells") and then identifying proteins with undetectable expression levels from either annotated protein expression (APE) or staining experiments where the reliability was classed as 'supportive'. Tumour expression was used to filter out proteins from C1-based networks by first filtering HPA tumour data (tumour = "urothelial cancer"). HPA reporting for tumours uses antibody based profiling for the 15,297 genes with anitbodies available, reporting expression levels (not detected, low, medium and high), the number of patients with this protein/expression level and the total number of patients for each tumour type. To filter C1 networks, proteins having undetectable expression levels across all patients were removed.

Proteins that were not expressed according to HPA, but were found in the relevant C1 or C3 proteome datasets were not filtered out; this reflects that HPA protein expression in urothelial/tumour cells may not exactly match that of our NHUC cells.

NetworKIN data import and processing

NetworKIN data downloaded and imported via the full tab-separated file (5th Feb 2016), comprised 459,749 rows specifying individual phosphosites on substrates, predicted kinases, SH2 domains, confidence scores and Ensembl identifiers [10].

Ensembl identifiers were mapped to UniProt accessions using UniProt ID mapping file from ftp site (below) on 18th June 2016. ftp://ftp.uniprot.org/pub/databases/uniprot/current_release/knowledgebase/idmapping/by_organism/

All subsequent SQL-based queries filtered any NetworKIN confidence scores ≤ 1 to keep only the ~50% best predicted substrate interactors resulting in 145,547 kinase and 135,396 SH2 interactions in the database. Assigned UniProt accessions for each phosphosite (in either QP or HC datasets) were matched by NetworKIN substrate identifier and the sequence similarity score between observed phosphosite and the site's sequence

annotated in NetworKIN (using Oracle SQL function UTL_MATCH.EDIT_DISTANCE_SIMILARITY). To reduce the number of irrelevant and/or non-informative matches returned, we further filtered here using HPA.

DIAMOnD runs

We used the script DIAMOnD.py (version 05/12/2014) with parameters n = 200 and alpha = 1 for differential analysis of C1 and C3 pathway enrichments. These parameter values are consistent with those suggested by DIAMOnD authors as sensible choices over a range of benchmark tests [11].

Database and scripting

We created a custom Oracle 12c database to import, clean and query datasets, with supporting scripts developed in R version 3.3; final network rendering and annotation used Cytoscape v3.4 [12].

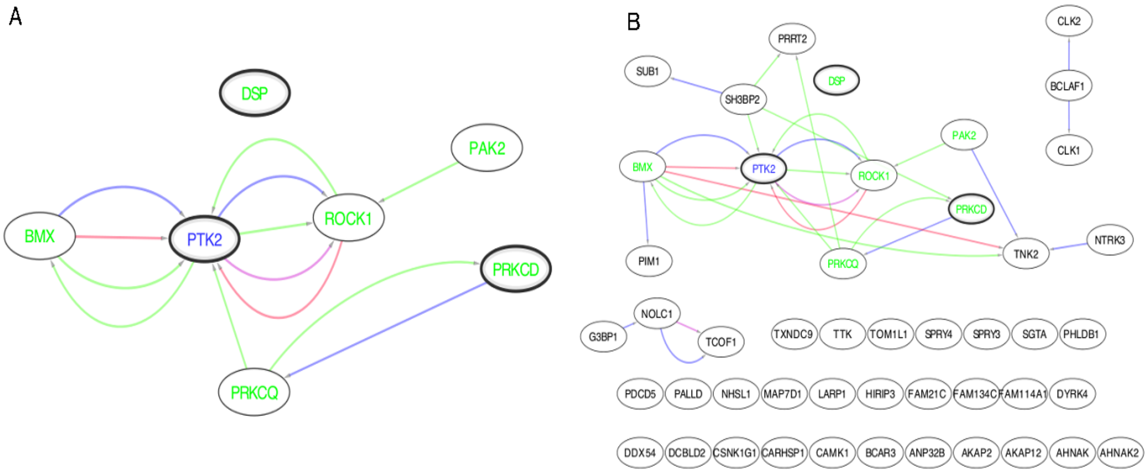

Illustration 2: Mapping of differential pathway Reactome seed proteins and non-Reactome proteins to the filtered effective network. (**A**) Seed proteins (example for C3 pathways 3 & 4) with interactions inferred from the filtered effective network. (**B**) Seed proteins and other proteins found from differential pathway analysis but not in Reactome are mapped onto the filtered effective network [Green/blue text: C3/shared seeds for pathway; bold outline: observed in QP and/or HC].

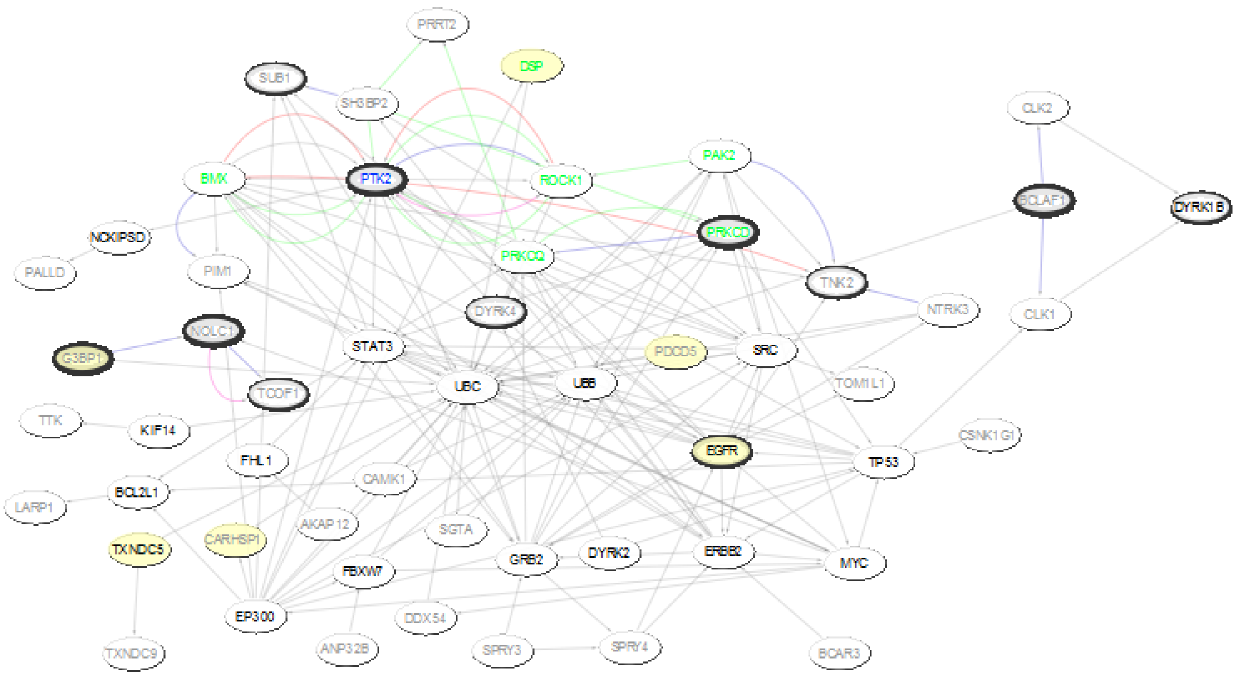

Illustration 3. Functional Interactome (FI) returned by using all differential pathway seeds, non-Reactome proteins and the linkers that connect them. Seed proteins are now functionally connected with the non-Reactome singletons (e.g. SPRY3, SGTA & BCAR3). Weakly connected proteins (i.e. of degree 1 such as BCAR3), although not informative here, are used to query the phosphoproteomics dataset for kinase-substrate interactions [Green/blue text: C3/shared seeds; light grey text: non-Reactome protein; (heavy) bold outline: observed in HC and/or QP; yellow fill: observed C3 proteome; edge colours for HSN as before.]

REFERENCES

1. Lombardi B, Rendell N, Edwards M, Katan M, Zimmermann JG. Evaluation of phosphopeptide enrichment strategies for quantitative TMT analysis of complex network dynamics in cancer-associated cell signalling. EuPA Open Proteom. 2015; 6:10–5.
2. Pinto G, Alhaiek AA, Amadi S, Qattan AT, Crawford M, Radulovic M, Godovac-Zimmermann J. Systematic nucleo-cytoplasmic trafficking of proteins following exposure of MCF7 breast cancer cells to estradiol. J Proteome Res. 2014; 13:1112–27.
3. Taus T, Köcher T, Pichler P, Paschke C, Schmidt A, Henrich C, Mechtler K. Universal and confident phosphorylation site localization using phosphoRS. J Proteome Res. 2011; 10:5354–62.
4. Rauniyar N, Gao B, McClatchy DB, Yates JR. Comparison of protein expression ratios observed by sixplex and duplex TMT labeling method. J Proteome Res. 2013; 12:1031–9.
5. di Martino E, L'Hôte CG, Kennedy W, Tomlinson DC, Knowles MA. Mutant fibroblast growth factor receptor 3 induces intracellular signaling and cellular transformation in a cell type- and mutation-specific manner. Oncogene. 2009; 28:4306–16.
6. Cerami EG, Gross BE, Demir E, Rodchenkov I, Babur O, Anwar N, Schultz N, Bader GD, Sander C. Pathway Commons, a web resource for biological pathway data. Nucleic Acids Res. 2011; 39:D685–90.
7. Bueno A, Morilla I, Diez D, Moya-Garcia AA, Lozano J, Ranea JA. Exploring the interactions of the RAS family in the human protein network and their potential implications in RAS-directed therapies. Oncotarget. 2016; 7:75810–26. https://doi.org/10.18632/oncotarget.12416.
8. Newman M. Networks: an introduction. Oxford University Press. 2010.
9. Wu G, Dawson E, Duong A, Haw R, Stein L. ReactomeFIViz: the Reactome FI Cytoscape app for pathway and network-based data analysis. F1000Res. 2014; 3:1–14.
10. Hornbeck PV, Zhang B, Murray B, Kornhauser JM, Latham V, Skrzypek E. PhosphoSitePlus, 2014: mutations, PTMs and recalibrations. Nucleic Acids Res. 2015; 43:D512–20.
11. Ghiassian SD, Menche J, Barabási AL. A DIseAse MOdule Detection (DIAMOnD) algorithm derived from a systematic analysis of connectivity patterns of disease proteins in the human interactome. PLOS Comput Biol. 2015; 11:e1004120.
12. Shannon P, Markiel A, Ozier O, Baliga NS, Wang JT, Ramage D, Amin N, Schwikowski B, Ideker T. Cytoscape: a software environment for integrated models of biomolecular interaction networks. Genome Res. 2003; 13:2498–504.

A

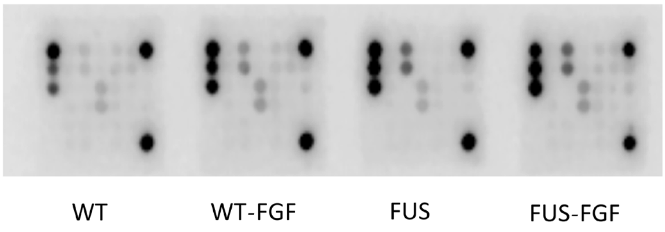

B

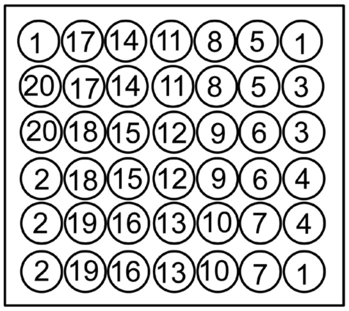

Position	Phosphoprotein	Modification
1	Positive Control	
2	Negative Control	
3	ERK1/2 (T202/Y204)	Phosphorylation
4	Stat1 (Y701)	Phosphorylation
5	Stat3 (Y705)	Phosphorylation
6	Akt (T308)	Phosphorylation
7	Akt (S473)	Phosphorylation
8	AMPKα (T172)	Phosphorylation
9	S6 Ribosomal Protein (S235/236)	Phosphorylation
10	mTOR (S2448)	Phosphorylation
11	HSP27 (S78)	Phosphorylation
12	Bad (S112)	Phosphorylation
13	P70 S6 Kinase (T389)	Phosphorylation
14	PRAS40 (T246)	Phosphorylation
15	p53 (S15)	Phosphorylation
16	p38 (T180/Y182)	Phosphorylation
17	SAPK/JNK ((T183/Y185)	Phosphorylation
18	PARP (D214)	Cleavage
19	Caspase-3 (D175)	Cleavage
20	GSK-3β (S9)	Phosphorylation

Supplementary Figure 1: Antibody-based array of phosphorylation profile of intracellular signalling proteins analyzed for four experimental conditions: WT, WTFGF, FUS and FUS-FGF. (**A**) Representative image of the antibody based arrays. (**B**) Schematic overview of the array with the indication of the antibodies to phosphoproteins spotted on it and their position.

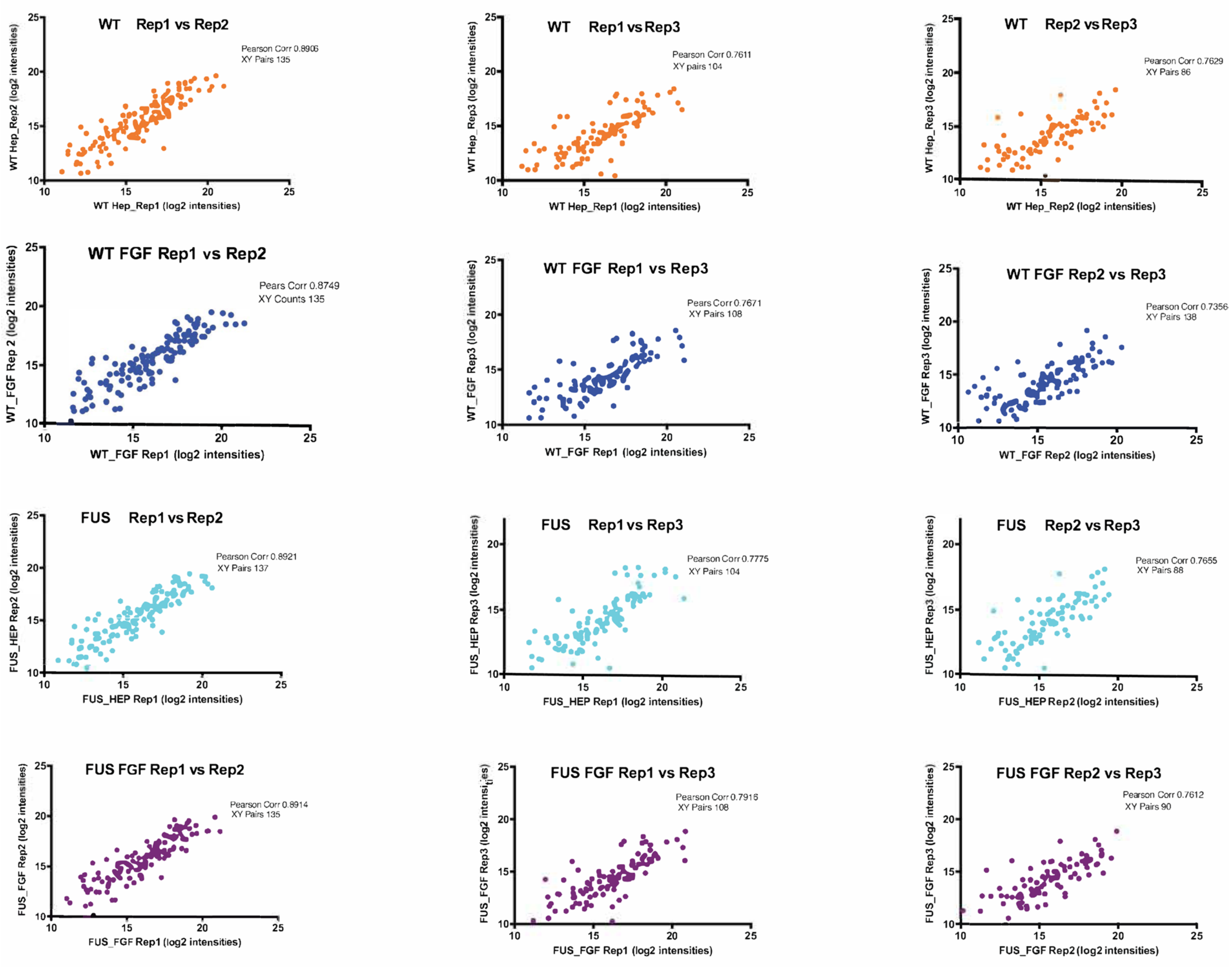

Supplementary Figure 2: Replicate correlations for phosphoproteomics data from four experimental conditions: WT, WT-FGF, FUS and FUS-FGF. The correlation of the intensities of the reporter ions measured in the three replicates is always high (Pearson coefficients ≥ 0.75).

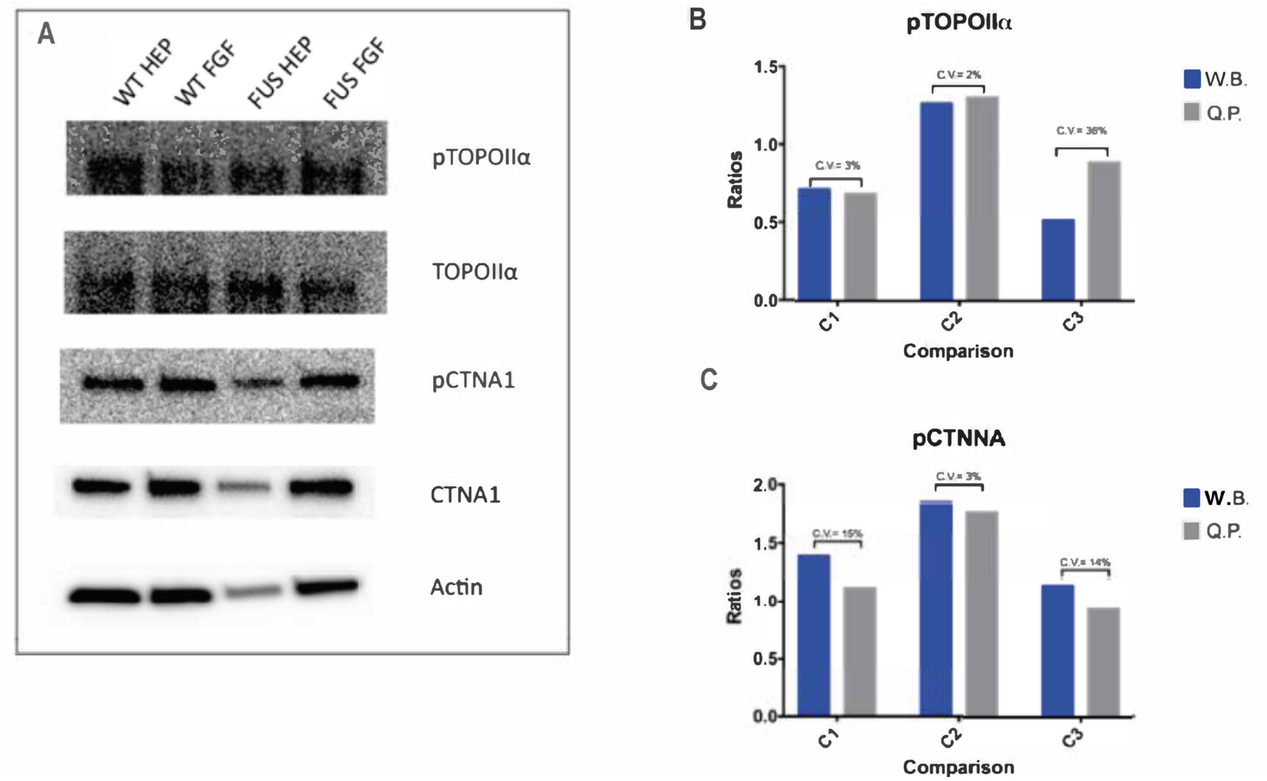

Supplementary Figure 3: Support for the accuracy of phosphosite quantification: comparison with data derived from Western blotting (**A**) Western blots obtained from the detection of phosphorylated and non-phosphorylated epitopes in CTNA1 and immunoprecipitated TOPIIα; β-actin was used as a loading control. (**B**) Densitometric analysis of signals detecting the phosphorylated fraction of the proteins has been used to obtain semi-quantitative information of the ratios measured in C1, C2 and C3 (Western blotting; W. B.) and the measurements compared with those obtained from the phosphoproteomic analysis (quantitative phosphoproteomics Q. P.); the agreement between the 2 measurements is good, with a coefficient of variation (C.V.) being below 15% (the only exception is the ratio calculated for pTOPIIα in C3 where the C.V. is 36%).

A

	HC Dataset	Dataset
Unique Phosphopeptides	523	752
Proteins	308 (16 exclusive)	382 (90 exclusive)
Total Counts for quantification	1647	2052

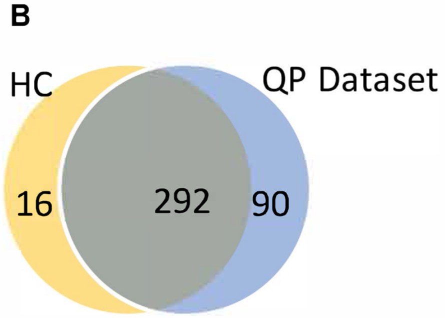

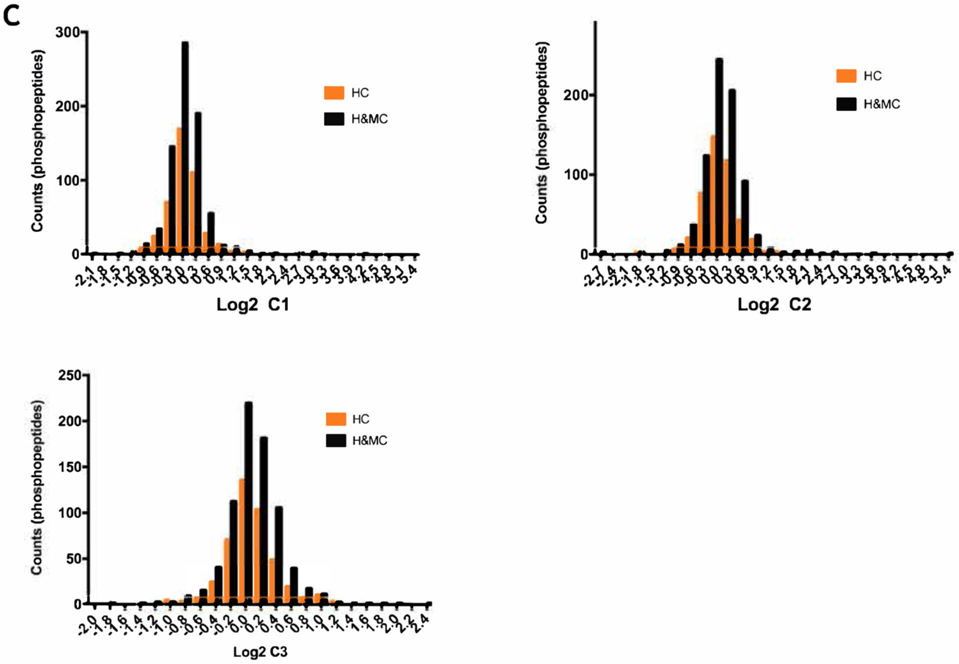

Supplementary Figure 4: HC and QP phosphoproteome datasets. The normalised phosphoproteomic dataset was filtered with two different criteria. The quantitative phosphoproteome data set (QP, Supplementary Table 1) includes all phosphopeptides identified with an FDR ≤ 0.05 and having a valid quantification for all four reporter ions/sample types (phosphosite localization has been manually inspected if the PhosphoRS probability was lower than 0.75). The high confidence quantitative phosphoproteome data set (HC, Supplementary Table 2) collects phosphopeptides identified with an FDR ≤ 0.01 and includes some phosphopeptides that were not observed and quantified in all four experimental samples. (**A**) Summary of identified phosphopeptides and annotated spectra in HC and QP datasets. (**B**) Shared and distinct HC and QP proteins (**C**) Distributions of HC (orange) and QP (black) $\log_2$ ratios for the three comparisons C1, C2 and C3.

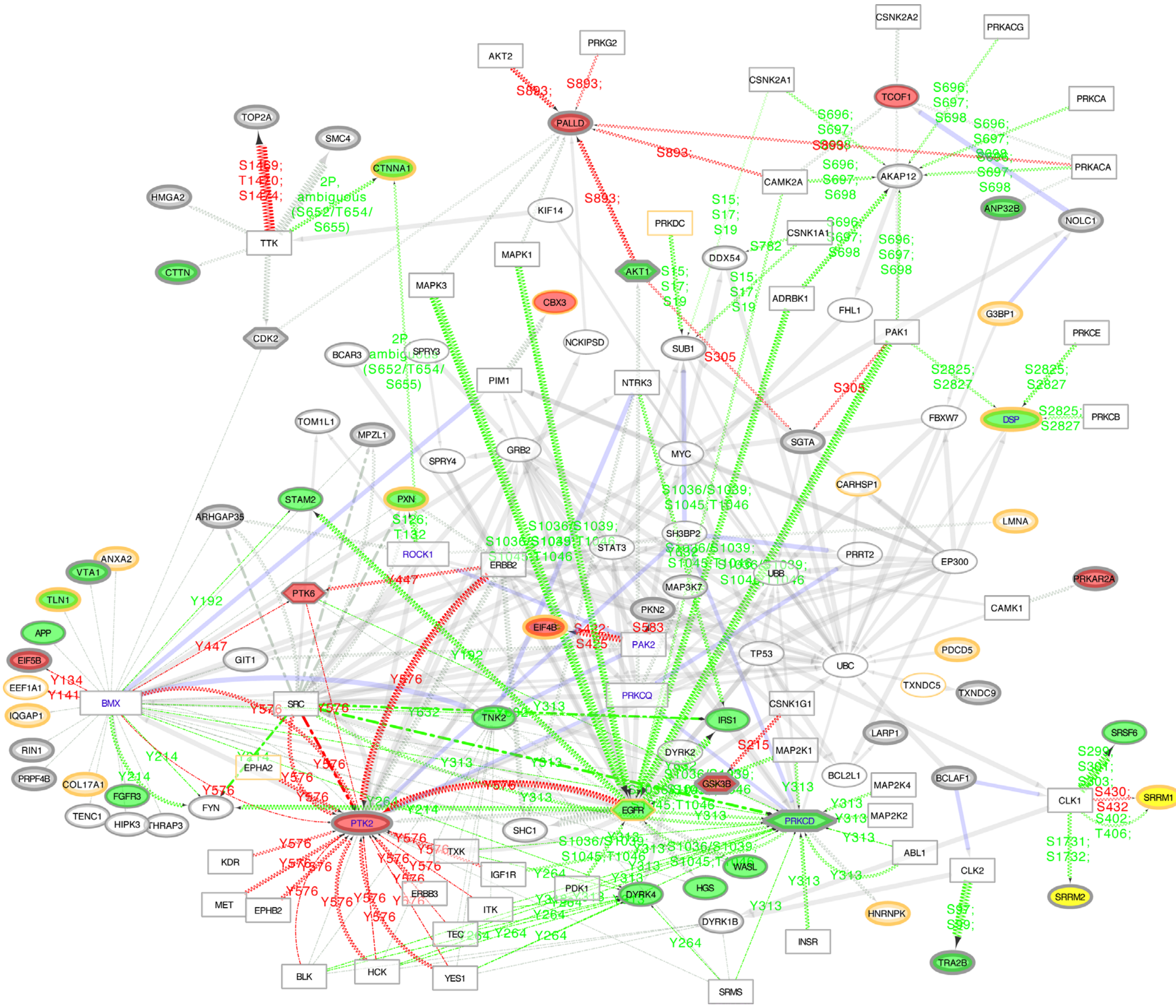

Supplementary Figure 5: Detailed functional interaction network for differentially regulated apoptotic cleavage/ execution phase pathways in FGF1-stimulated cells expressing WT FGFR3 IIIb. Changes in signalling pathways in WT FGFR3b expressing cells, highlighted by the differential analysis, are from 'Apoptotic cleavage of cellular proteins' (R-HSA- 111465) and its parent pathway 'Apoptotic execution phase' (Reactome ID: R-HSA- 75153) (p < 0.01, FDR <1%). Although not the highest ranked C3-specific pathways from Reactome enrichment analysis, both met inclusion threshold (FDR <1%) and included several proteins with experimental evidence: PTK2, DSP and PRKCD (in QP and HC datasets). Significantly altered quantitative phosphorylation ratios for C3 are marked as either up (red) or down (green wavey lines) with corresponding phosphosite labelled (or multiple labels for ambiguous sites). Protein confidence: Predicted/inferred (thin grey border), QP (medium grey border), HC (thick grey border) and in proteome (orange border). Protein change w.r.t C1: red fill (protein contains at least on phosphosite up-regulated in C3 compared to C1), green (down regulated compared to C1), yellow (mixed up/down phosphosites on protein). Protein shape: ellipse (substrate), rectangle (kinase), hexagon (kinase and substrate). Note protein-SH2 interactions as dashed lines.

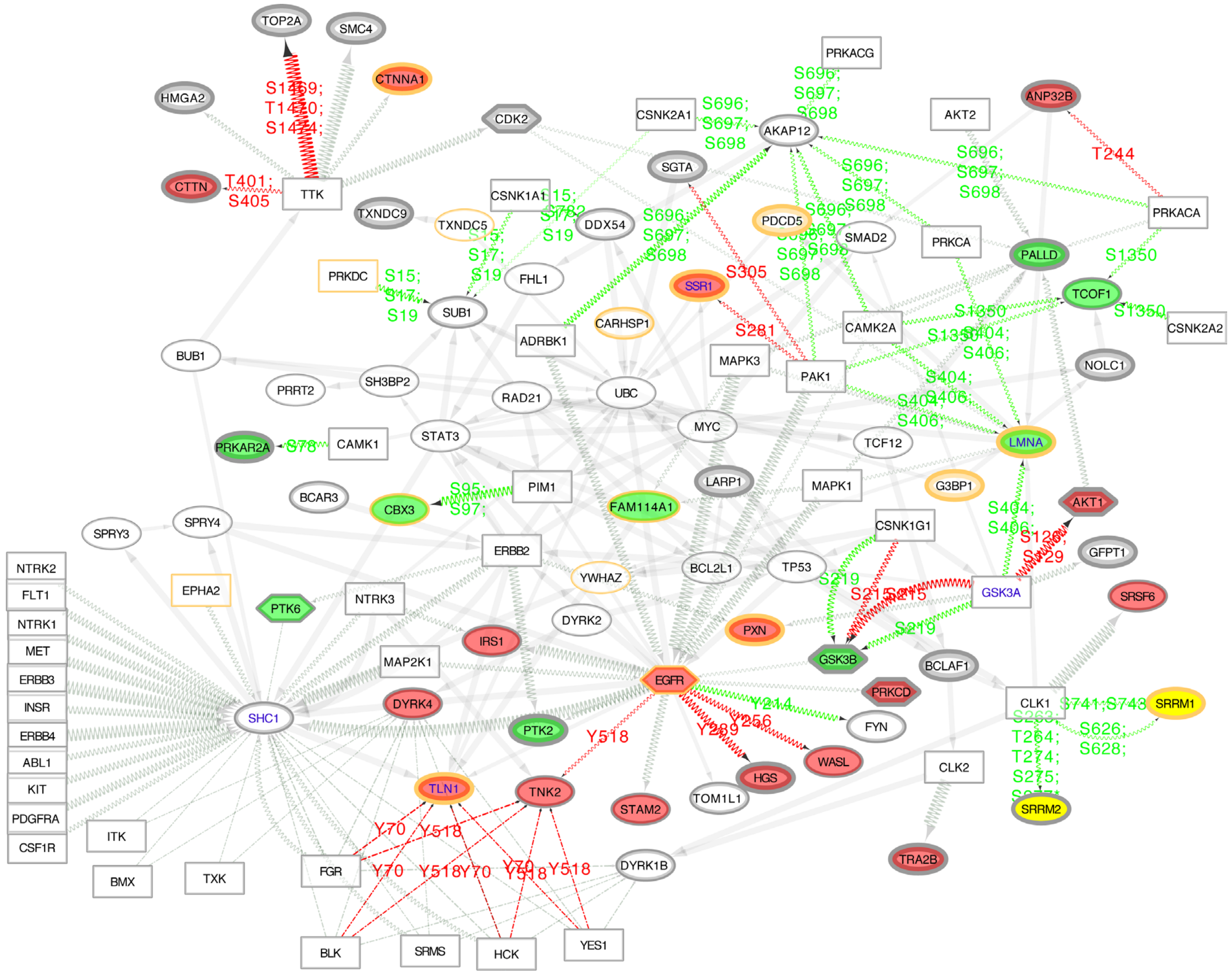

Supplementary Figure 6: Detailed functional interaction network for differentially regulated chaperone pathways in cells expressing FGFR3-TAAC3 fusion protein. 'XBP1(S) activates chaperone genes' (Reactome ID: R-HSA-381038) and its parent pathway 'IRE1alpha activates chaperones' (Reactome ID: R-HSA-381070) ($p < 0.01$, FDR <1%) have been identified from C1-specific alterations in QP & HC proteins LMNA, TLN1 and SSR1, plus network-related proteins SHC1 and GSK3A. Seed proteins (blue text) form a core network that has been expanded to include neighbouring interactions by overlaying other C1 differential proteins, likely kinase/SH2-substrate interactions, local neighbours on the human signalling network and predicted functional interactions from ReactomeFI. Significantly altered quantitative phosphorylation ratios for C1 are marked as either up (red) or down (green wavey lines) with corresponding phosphosite labelled (or multiple labels for ambiguous sites). Protein confidence: Predicted/inferred (thin grey border), QP (medium grey border), HC (thick grey border) and in proteome (orange border). Protein change w.r.t C3: red fill (protein contains at least on phosphosite up-regulated in C1 compared to C3), green (down regulated compared to C3), yellow (mixed up/down phosphosites on protein). Protein shape: ellipse (substrate), rectangle (kinase), hexagon (kinase and substrate). Note protein-SH2 interactions as dashed lines.

Supplementary Table 1: Table summarizing the QP dataset. The first sheet, lists the unique phosphopeptides identified and quantified based on the 3 replicates. Peptides identified with the same phosphosites have been grouped together and quantification of each site has been obtained based on the averaged intensities measured in each sample for each peptide belonging to the same group. The second and the third sheets collect the raw data obtained after TiO_2 enrichment (second sheet) and anti-pY IP (third sheet): indication of the phosphopeptides that have been grouped together and all the relevant information linked to the peptide identification and phosphosite localization are present. See Supplementary_Table_1

Supplementary Table 2: Table summarizing the HC-QP dataset. For each phosphopeptide, identified with a FDR ≤ 0.01, the averaged intensity measured in each replicate and in each sample is reported together with the total number of spectra used to obtain the reported values. Ratios calculated for C1, C2 and C3 are also here reported and also their $\log_2$ transformation. See Supplementary_Table_2

Supplementary Table 3: Table summarizing the reference proteome. Sheet1, sheet2 and sheet3 summarize the identified and quantified proteins respectively in C1, C2 and C3 with the indication of ratios measured in each replicate, their $\log_2$ transformation and the mean values obtained. Subsequent sheets summarize all the individual datasets used to obtain the reference proteomes for C1, C2 and C3. See Supplementary_Table_3

Supplementary Table 4: Table summarizing phosphosites having a $\log_2$ ratio (C1, C2 or C3), reported in the HC-QP $\geq$ 0.5 or $\leq$ –0.5. The observed ratios, the number of spectra used to compute the ratios and the number of replicates (1, 2 or 3) where the phosphopeptides have been observed, are also reported. See Supplementary_Table_4

Supplementary Table 5: Tables summarizing Sig B positive phosphopeptides in C1, C2 and C3 with the indication of the associated *p*-Value. See Supplementary_Table_5

Supplementary Table 6: Table summarizing network seeds for C1 and C3

Protein	C1 HC phosphosites	C1 mean quant ratio	C1 QP network seed?	C3 HC phosphosites	C3 mean quant ratio	C3 QP network seed?
**AHNAK**	S135	2.06	Y	S135	2.33	Y
**CTR9**	S1016; S1020; S1021	0.50	Y	S1016; S1020; S1021	0.51	Y
**CTR9**	S1081; S1085	0.66	Y			Y
**CTTN**	T401; S405	1.56	Y			
**DCBLD2**	T722; S724	0.53	Y			
DKC1	S513	0.66	*(HC only)*			
**EIF4G1**	S1596	1.80	Y	S1596	1.72	Y
**FAM21C**	S284; T287	1.72		S284; T287	2.02	Y
**HGS**	Y132	35.63	Y			
**HLCS**				S79	1.44	Y
**NOLC1**	S332; S333	0.39	N			Y
**PDCD5**	S119	1.73	Y	S119	1.72	Y
**PRKCD**				Y313	0.69	Y
PRPF4B				?	0.63	*(HC only)*
**SGTA**	S301; S305	1.78	Y	S301; S305	2.10	Y
**SRRM2**			Y	S1404; T1413; S1415	1.43	Y
**TLN1**	Y70	2.65	Y			
**TOP2A**	S1469; T1470; S1474	0.68	Y			Y
**TRA2B**				S97; S99	0.51	Y
**TRA2B**				S97; S99; S101	0.57	Y
ABCF1, AHNAK2, AKAP2, AKT1, ANP32B, APP, CARHSP1, CBX5, CHMP2B, CPSF2, FGFR3, G3BP1, HDAC2, HDGFRP2, HNRNPD, IWS1, LARP1, LMNA, PRKAR2A, RFC1, RPLP1, SCRIB, SEPT2, SORBS3, SRPR, SSB, SSR1, STK10, TCOF1, THUMPD1, TMPO, TNK2, TOM1L1, UTP18, VTA1, WASL			**C1 seeds only in QP dataset**			
ACBD5, ANXA2, BABAM1, BCAR3, CTNNA1, DHX16, DSP, DYRK4, EGFR, EIF3C, FAM114A1, FAM134C, GTF2F1, HIRIP3, IRS1, KLRB1, MAP7D1, NCAPG, NDRG1, NHSL1, NOLC1, PALLD, PFDN4, PTK2, PTK6, PTPN12, PXN, SRSF6, STAM2, SUDS3, TRIP10, TXNDC9, UFL1, WNK1						**C3 seeds only in QP dataset**
AKAP12, ARHGAP5, BCLAF1, CBX3, COL17A1, CTNND1, CTPS1, DDX54, EEF1A1, EIF4B, FYN, GSK3B, HDAC1, HMGA1, KTN1, PDAP1, PEA15, PHLDB1, PKN2, PRPF40A, SNW1, SRRM1, SRRM2, SRSF2, SUB1, TNKS1BP1			**C1 & C3 common QP seeds**			**C1 & C3 common QP seeds**

Protein seeds for C1 and C3 are derived from QP and overlap with HC as indicated. Seeds only identified using QP are shown in lower 3 rows.
